# Systemic immune challenges trigger and drive Alzheimer-like neuropathology in mice

**DOI:** 10.1186/1742-2094-9-151

**Published:** 2012-07-02

**Authors:** Dimitrije Krstic, Amrita Madhusudan, Jana Doehner, Prisca Vogel, Tina Notter, Claudine Imhof, Abigail Manalastas, Martina Hilfiker, Sandra Pfister, Cornelia Schwerdel, Carsten Riether, Urs Meyer, Irene Knuesel

**Affiliations:** 1 Institute of Pharmacology and Toxicology, University of Zurich, Winterthurerstrasse 190, CH-8057, Zurich, Switzerland; 2 Department of Clinical Research, Tumor Immunology, University of Bern, Murtenstrasse 35, Bern, Switzerland; 3 Physiology and Behavior Laboratory, ETH Zurich, Schorenstrasse 16, 8603, Schwerzenbach, Switzerland

**Keywords:** Mouse model of sporadic Alzheimer`s disease, Aging, Immune challenge, Systemic infection, Neuroinflammation, Cytokines, Interleukins, PolyI:C

## Abstract

**Background:**

Alzheimer’s disease (AD) is the most prevalent form of age-related dementia, and its effect on society increases exponentially as the population ages. Accumulating evidence suggests that neuroinflammation, mediated by the brain’s innate immune system, contributes to AD neuropathology and exacerbates the course of the disease. However, there is no experimental evidence for a causal link between systemic inflammation or neuroinflammation and the onset of the disease.

**Methods:**

The viral mimic, polyriboinosinic-polyribocytidilic acid (PolyI:C) was used to stimulate the immune system of experimental animals. Wild-type (WT) and transgenic mice were exposed to this cytokine inducer prenatally (gestation day (GD)17) and/or in adulthood. Behavioral, immunological, immunohistochemical, and biochemical analyses of AD-associated neuropathologic changes were performed during aging.

**Results:**

We found that a systemic immune challenge during late gestation predisposes WT mice to develop AD-like neuropathology during the course of aging. They display chronic elevation of inflammatory cytokines, an increase in the levels of hippocampal amyloid precursor protein (APP) and its proteolytic fragments, altered Tau phosphorylation, and mis-sorting to somatodendritic compartments, and significant impairments in working memory in old age. If this prenatal infection is followed by a second immune challenge in adulthood, the phenotype is strongly exacerbated, and mimics AD-like neuropathologic changes. These include deposition of APP and its proteolytic fragments, along with Tau aggregation, microglia activation and reactive gliosis. Whereas Aβ peptides were not significantly enriched in extracellular deposits of double immune-challenged WT mice at 15 months, they dramatically increased in age-matched immune-challenged transgenic AD mice, precisely around the inflammation-induced accumulations of APP and its proteolytic fragments, in striking similarity to the post-mortem findings in human patients with AD.

**Conclusion:**

Chronic inflammatory conditions induce age-associated development of an AD-like phenotype in WT mice, including the induction of APP accumulations, which represent a seed for deposition of aggregation-prone peptides. The PolyI:C mouse model therefore provides a unique tool to investigate the molecular mechanisms underlying the earliest pathophysiological changes preceding fibrillary Aβ plaque deposition and neurofibrillary tangle formations in a physiological context of aging. Based on the similarity between the changes in immune-challenged mice and the development of AD in humans, we suggest that systemic infections represent a major risk factor for the development of AD.

## Background

Neuroinflammation was one of the prominent pathological features described by Alois Alzheimer in his first case report in 1907. Today we know that specific markers of neuroinflammation are selectively enriched in brain areas affected by Alzheimer’s disease (AD) neuropathology [1], and that individuals with high plaque burden without dementia show virtually no evidence of neuroinflammation [2]. This is further supported by positron emission tomography (PET) imaging studies, which have shown that cognitive status is inversely correlated with microglial activation in patients with AD [3]. In addition, recent genome-wide association studies have identified significant correlations between components of the innate immune system and the incidence of sporadic AD [4], supporting the link between the immune system and AD pathophysiology suggested by previous retrospective epidemiological studies in humans [5,6].

However, the precise role of neuroinflammation in the disease etiology is still controversial, ranging from representing a possible cause to being a by-product of the disease [7] or even being beneficial [8]. In 1996 it was proposed that interleukin (IL)-1, an inflammatory cytokine whose levels are increased in the brains of patients with AD [9], may represent a driving force in the pathogenesis of AD [10,11]. Although this hypothesis was in accordance with a meta-analysis of 17 epidemiological studies indicating that non-steroidal anti-inflammatory drugs might decrease the risk of developing AD [12], subsequent randomized trials not only failed to show a beneficial effect of anti-inflammatory drugs on the etiology of AD [13,14], but in fact found an increase in AD incidence in patients with mild cognitive impairment treated with these drugs as compared to placebo [15]. The resolution of apparent inconsistency came only recently with the revision of the Alzheimer`s Disease Anti-inflammatory Prevention Trial (ADAPT) hypothesis that supports a beneficial role of anti-inflammatory drugs only in the early, asymptomatic, phases of the disease [16]. However, *in vivo* experimental evidence to support an early and potentially causative role for systemic infections and neuroinflammation in the etiology of sporadic AD is still missing.

To elucidate the early role of inflammatory processes in the development of AD-like pathology in mice, we used the viral mimic polyriboinosinic-polyribocytidilic acid (PolyI:C), a synthetic analog of double-stranded RNA, to stimulate the immune system of our experimental animals [17,18]. We have previously shown that a single exposure to PolyI:C during late gestation triggers the expression of several inflammatory cytokines in the fetal brain [19], evokes a reduction in adult neurogenesis, accompanied by memory impairments [19,20], and accelerates protein depositions in the hippocampus of the adult offspring [21]. In the current study, we tested the hypothesis that the prenatal immune challenge during late gestation results in pathological aging, and predisposes the offspring to aging-associated AD-like neuropathology and cognitive decline [22]. In addition, we tested the effects of systemic immune challenge in adulthood on the progression of the AD-like phenotype either in prenatally challenged wild-type (WT) mice or in transgenic AD (3xTg-AD) mice [23].

## Methods

### Animals

All experimental procedures were approved by the local authorities of the Cantonal Veterinary Office in Zurich and carried out in agreement with the Principles of Laboratory Animal Care (National Institutes of Health publication number 86–23, revised 1985).

Animals (see Table 1 for a complete list) were housed in groups of three to four in an optimized in-house hygiene area (University of Zurich Irchel, Zurich, Switzerland) or standard housing conditions in the Laboratory of Behavioural Neurobiology (ETH Zurich, Schwerzenbach, Switzerland) under a 12 hour light/dark cycle, with access to food and water *ad libitum*. Transgenic (3xTg-AD; encoding APP_swe_, and TauP301L on a homozygous PS1M146V knock-in background [23]) and the non-transgenic mice with the same genetic background (129/C57Bl6) were obtained from Dr Frank LaFerla, University of California, Irvine, CA, USA. WT C57BL/6 J mice were obtained from the breeding facility of the Institute of Laboratory Animal Science, University of Zurich (LTK Fuellinsdorf).

**Table 1:** Overview of the experimental animals used in this study

**Strain**	**Genotype**	**Animals, n**	**Treatment**		**Time of tissue collection (use)**	**Experiment**
			**Prenatal**	**Adult**		
129/C57Bl/6	Non-Tg	4 NaCI, 4 PolyI:C	–	4 months	15 months (IHC)	Systemic infection in TgAD mice
	3xTg-AD	5 NaCI, 7 PolyI:C	–	4 months	15 months (IHC)	
C57Bl6/J	Non-Tg	5 NaCI, 5 PolyI:C	GD17	–	3 weeks (E)	Prenatal infection in non-Tg mice: longitudinal changes in cytokine and brain APP/pTau levels and cognitive performance
		4 NaCI, 4 PolyI:C	GD17	–	3 months (IB/E)	
		5 NaCI, 5 PolyI:C	GD17	–	5 months (E)	
		16 NaCI, 15 PolyI:C	GD17	–	5 months (BT)	
		4 NaCI, 4 PolyI:C	GD17	–	6 months (IB/E)	
		6 NaCI, 6 PolyI:C	GD17	N*	12 months (IB)	
		4 NaCI, 5 PolyI:C	GD17	–	15 months (IB/E)	
		6 NaCI, 6 PolyI:C	GD17	–	15 months (IHC)	
		18 NaCI, 13 PolyI:C	GD17	–	20 months (BT)	
C57Bl6/J	Non-Tg	6 NN*, 6 NP, 6N*, 7 PP	GD17	9 months	12 months (IB)	Prenatal and adult infection in non-Tg mice
		10 NP, 11 PP	GD17	12 months	15 months (IHC)	
		6 NN, 5 NP, 4 PN, 5 PP	GD17	15 months	18 months (IHC)	

Abbreviations: APP, amyloid precursor protein; BT, behavioral testing; E, ELISA; GD, gestational day; IB, immunoblotting; IHC, immunohistochemistry; N = NaCI (0.9% saline); NN, prenatal and adult NaCI treatment; NP, prenatal NaCI and adult PolyI:C treatment; PN, prenatal PolyI:C and adult NaCI treatment; PP, prenatal and adult PolyI:C treatment; pTau, phosphorylated Tau.

*Control group of first cohort used in experiment 3.

### Polyriboinosinic-polyribocytidilic acid injections

Pregnant mouse dams of the C57Bl/6 J strain were given a single intravenous injection of 5 mg/kg PolyI:C potassium salt (P9582, 50 mg; Sigma-Aldrich Chemie GmbH, Buchs, Switzerland) dissolved in 0.9% saline (NaCl) with an injection volume of 5 ml/kg body weight or an equivalent volume of saline at gestation day (GD) 17. The animals were mildly restrained during the injection procedure using an acrylic mouse restrainer, and after the injection, were immediately placed back in their home cage and left undisturbed until the first cage change at 1 week after delivery.

### Behavioral testing

To assess a putative cognitive decline in aged mice exposed to a prenatal immune challenge, adult (5 months) and aged (20 months) mice were tested in the elevated Y-maze spontaneous alternation task (Table 1). This task is used to measure spatial recognition memory that depends on proper hippocampal functions, as confirmed by the severe memory impairment after excitotoxic lesion of this brain region [24]. It is based on the innate tendency of rodents to explore novel environments, that is, their preference to investigate a new arm of the maze rather than returning to one that was previously visited. This paradigm avoids the effects of other methods such as punishment (such as electric shock) or reward (such as food following phases of deprivation) that are commonly used in other paradigms and may have non-specific effects on the results. In addition, it does not require learning of a rule, thus it is useful for studying performance in rodents, particularly in aged animals.

#### Apparatus

The Y-maze apparatus was made of transparent Plexiglas and consisted of three identical arms (500 mm long × 90 mm wide) surrounded by transparent Plexiglas walls 100 mm in height. The three arms radiated from a central triangle (80 mm on each side) and were spaced 120 degrees from each other. The floor of the maze was covered with sawdust bedding, which was changed between each test run. The maze was elevated 900 mm above the floor, and was positioned in a well-lit room enriched with distal spatial cues. A digital camera was mounted above the Y-maze apparatus. Images were captured at a rate of 5 Hz, and transmitted to a PC running the EthoVision tracking system (Noldus Information Technology), which calculated the total distance moved and the number of entries into the three arms and the center zone of the Y-maze.

#### Procedure

Mice were placed in the center of the Y maze. An observer in an adjacent room viewed the mice through a video camera, and recorded the number and sequence of arm entries (defined as entry of the whole body into an arm) during a period of 5 minutes. Alternation was defined as entry into the three arms in any non-repeating order (for example, ABC, BAC, CBA). The percentage alternation was calculated as the total number of alternations divided by the possible alternations given the number of arm entries (total number of arm entries, 2). In addition to the analysis of percentage alternation, the total distance moved and the total number of arm entries were recorded and analyzed to assess general activity during the 5-minute test period.

### Mouse brain-tissue preparation

The tissue was fixed by perfusion and processed as described previously [21]. Briefly, animals were deeply anesthetized by intraperitoneal injection of pentobarbital (4 ml/kg body weight; Nembutal™; Lundbeck Inc., Deerfield, IL, USA) and perfused transcardially with PBS (pH 7.4), followed by 4% paraformaldehyde in PBS (Sigma-Aldrich). The brains were post-fixed for 4 hours at 4°C followed by cryoprotection for 24 hours in 30% sucrose. Randomly sampled serial sections (cut at 40 μm) were collected throughout the hippocampal formation starting at bregma level −0.82 to −3.64 [25], and stored at −20°C in cryoprotectant solution (50 mmol/l sodium phosphate buffer, pH 7.4, containing 15% glucose and 30% ethylene glycol; Sigma-Aldrich) until immunohistochemical or histological evaluation.

### Immunohistochemistry

All the free-floating sections were treated for 10 minutes with pepsin (Cat-No S3002; Dako, Carpinteria, CA, USA) 0.15 mg/ml in 0.2 N HCl at 37°C, to unmask epitopes and reduce non-specific staining [26,27]. Sections were briefly washed with PBS (pH 7.4). The pepsin-treated brain sections were then incubated overnight at 4°C in the primary antibody solutions diluted in PBS (see Table 2 for details) containing 2% normal goat serum and 0.2% Triton X-100.

**Table 2:** Summary of antibodies used in this study

**Antibody**	**Manufacturer**	**Description/Nr**	**Dilution**	**Application**
β-Actin	Millipore, Billerica, MA, USA	Mouse monoclonal, clone 4, MAB1522	1:20,000	IB
Amyloid-β_1–16_	Covance, Princeton, NJ, USA	Mouse monoclonal, clone 6E10, SIG-39320	1:1,000	IHC/IB
Amyloid-β_1–40/42_	Millipore	Rabbit polyclonal, AB5076	1:2,000	IHC
APP (A4), N-term	Millipore	Mouse monoclonal, clone 22 C11, MAB348	1:2,000	IHC/IB
APP, C-term	Sigma-Aldrich Chemie GmbH, Buchs, CH	Rabbit polyclonal, A8717	1:4,000	IB
Cleaved Caspase-6	Cell Signaling Technology, USA	Rabbit polyclonal, Nr. 9751	1:500	IHC
CD68	AbD Serotec Ltd, Oxford, UK	Rat monoclonal, MCA341R	1:2,000	IHC
GFAP	Dako Schweiz AG, Baar, CH	Rabbit polyclonal, Z334	1:5,000	IHC
pTau^T205^	Abcam, Cambridge, UK	Rabbit polyclonal, ab4841	1:1,000	IHC/IB
PHF-Tau	Thermo Fisher Scientific, Fremont, CA, USA	Mouse monoclonal, MN1060	1:1,000	IB
Tau-Ab2	Thermo Fisher Scientific	Mouse monoclonal, total Tau, clone Tau-5	1:4,000	IB

APP, amyloid precursor protein; GFAP, glial fibrillary acidic protein; IB, immunoblotting; IHC, immunohistochemistry; pTau, phosphorylated Tau; PHF, paired helical filament.

After three washes in PBS, tissue sections processed for immunoperoxidase labeling were incubated for 30 minutes at room temperature in biotinylated secondary antibodies (diluted 1:500; Jackson ImmunoResearch Laboratories Inc. West Grove, PA, USA), followed by three rinses in PBS. A commercial kit (Vectastain Kit; Vector Laboratories; Burlingame, CA, USA) was then used with 3,3-diaminobenzidine (DAB; Sigma–Aldrich Inc.), and sections were stained for 5 to 10 minutes [21]. After three washes in PBS, sections were mounted onto gelatinized glass slides and air-dried overnight. The sections were then dehydrated through ethanol, cleared in xylene, mounted with resinous mounting medium (Eukitt^TM^™; Sigma-Alrich), and coverslipped.

For immunofluorescence staining, sections were incubated for 30 minutes at room temperature with secondary antibodies coupled to Alexa488 (diluted 1:1000; Molecular Probes, Eugene, OR, USA), Cy3 (diluted 1:500; Molecular Probes) or Cy5 (diluted 1:200; Jackson ImmunoResearch Laboratories).

For the visualization of β-sheet enriched proteinous aggregates, sections were counterstained with thioflavinS (Sigma-Aldrich), involving incubation for 10 minutes in filtered 0.1% aqueous thioflavinS solution at room temperature, followed by two washes for 5 minutes each in 80% EtOH, one wash for 5 minutes in 95% ethanol, and three washes for 5 minutes each in distilled water. Brain sections were air-dried in the dark and mounted with aqueous permanent mounting medium (Dako) containing 1.5 μg/ml DAPI (Thermo Scientific (Schweiz) AG, Reinach, Switzerland).

Double‐or triple‐labeling was visualized by confocal microscopy (LSM-710; Zeiss, Jena, Germany) using 40× (numerical aperture (NA) 1.3) and 63× (NA 1.4) objectives and sequential acquisition of separate channels. Z-stacks of consecutive optical sections (6 to 12; 1024 × 1024 pixels, spaced 0.5 to 1 μm in *z*) were summed and projected in the *z* dimension (maximal intensity), and merged using the image analysis software Imaris (Bitplane, Zurich, Switzerland) for visual display. Cropping of images, adjustments of brightness and contrast were performed using Adobe Photoshop and were identical for each staining.

### Fluoro-Jade and silver staining

For the detection of dystrophic neurites and proteinous aggregates in immune-challenged 3xTg-AD mice, we used two commercial kits (Fluoro-Jade® Kit (Millipore, Billerica, MA, USA, catalog number AG325) and FD NeuroSilver^TM^™; Kit II (FD NeuroTechnologies Inc, Ellicott City, MD, USA).

For the Fluoro-Jade staining, tissue sections processed for anti-Aβ immunofluorescence staining were mounted on gelatin-coated slides and air-dried for 2 to 3 hours, then stained in accordance with the manufacturer’s protocol. In brief, slides were immersed in a solution of 1% sodium hydroxide in 80% ethanol for 5 minutes. Next, they were washed in 70% ethanol and distilled water for 2 minutes each. This was followed by 10 minutes incubation in a solution of 0.06% potassium permanganate. After rinsing the slides in distilled water, they were incubated in the staining solution for 20 minutes (dye concentration: 0.0004%). The slides were washed three times in distilled water before air-drying for 1 hour. After immersion in xylene, slides were coverslipped with aqueous mounting medium containing DAPI (Dako, Glostrup, Denmark.

For the silver staining, free-floating perfusion-fixed brain slices (40 μm thick) prepared for normal immunohistochemistry, were processed in accordance with the manufacturer’s instructions. For both procedures, control sections obtained from adult WT mice that had received unilateral intra-hippocampal injections of kainic acid (a manipulation that results in rapid degeneration of hippocampal interneurons and CA1 pyramidal cells [28]) served as controls.

### Quantification of immunohistochemical staining

All immunoperoxidase-stained slides were scanned with an automated upright slide-scanning microscope (Mirax Midi Slide Scanner; Zeiss) in bright-field mode. Images were acquired with a digital camera (1288 × 1040 pixels, with a pixel size of 0.23 μm; AxioCam monochrome charge-coupled display; Zeiss) with a 20× objective (NA 0.8) using the software Pannoramic Viewer (version 1.8.3; 3D Histech Ltd, Budapest, Hungary). A densitometric segmentation analysis was performed for the anti-Aβ (15 months, prenatal infection time course), anti-phosphorylated (p)Tau (hilar mossy cells, 15 months, prenatal infection), anti-glial fibrillary acidic protein (GFAP) (astrocytes, double immune-challenge experiment), and anti-CD68 (activated microglia, double immune-challenge experiment) immunoperoxidase stains to measure the relative percentage and the mean size of the labeled cells covered by the immunoreactive signals in the hippocampal formation. Here, the immunoreactivity (IR) in the neuropil served as the reference for non-specific background staining.

The quantitative analyses were performed on one randomly selected hemisphere and the researcher was blinded to the genotype/treatment of the animals. Hippocampi from four to six brain sections per animal were outlined, and the positively labeled cells identified by using a segmentation threshold with Histoquant software (3D Histech Ltd, Budapest, Hungary). The overall area and mean size of all segmented cells were calculated and divided by the total area of the outlined region. For the anti-APP and pTau IR in axonal and dendritic regions (experiments involving mice exposed to a prenatal infection and a double immune-challenge), a similar procedure was applied using the standard threshold algorithm of ImageJ software (version 1.45b; NIH, Bethesda, MD, USA) applied to exported high-magnification TIFF images (40x) from the digital Pannoramic Viewer. Pixel brightness was measured in several disease-relevant hippocampal subregions, including CA1 stratum pyramidale, radiatum, lacunosum moleculare, dentate gyrus (DG) inner and outer molecular layer, neuropil in the hilus, CA3 stratum pyramidale, and lucidum, and in the corpus callosum (serving for background correction). For the double immune-challenged mice and their controls, one series of randomly sampled brain sections (n = 6, sampling fraction 1/12) processed for anti-Aβ_1–40/42_ immunohistochemistry was used to stereologically estimate the numerical density of Aβ deposits in the hippocampus proper, the lateral entorhinal cortex, and neocortical areas (somatosensory cortex). The volumes were estimated by the formula

$$\text{V}=\sum A\times t_{\text{nom}}\times 1/ssf$$

where ∑*A* = the summed area of the brain region, *t*_nom_ = the nominal section thickness, and *ssf* = the section sampling fraction. Aβ plaques, identified by their fibrillary morphology, were counted exhaustively within the corresponding areas, using the marker counting tool of Histoquant software. The total number of plaques (*P*_tot_) and the numerical density (*P*_tot/mm3_) were estimated by the formula

$$P_{\text{tot}/\text{mm}3}=\sum P\times 1/\text{ssf}\times {\text{mm}}^{-3}$$

where ∑*P* is the sum of plaques. Densitometric and stereological values were averaged per animals and used in the statistical analyses.

### Processing of human post-mortem tissue

Paraffin wax-embedded brain sections (4 μm thick) of the temporal lobe obtained from a 88-year-old patient diagnosed with AD (generously provided by Professor Dr Manuela Neumann, Institute of Neuropathology, University of Zurich, Zurich, Switzerland) were dewaxed in xylene (3 × 3 minutes) and rehydrated in decreasing concentrations of ethanol (2 × 100%, 96%, 70% ethanol, and 2 × H_2_O for 3 minutes each) followed by washing for 5 minutes in 50 mmol/l Tris-buffered saline (pH 7.4). Optimal antigen retrieval was achieved by treating sections for 5 minutes with 95% formic acid (involving the anti-amyloid-β_1–40/42_ antibody, 1:200, AB5076; Millipore) or a citrate buffer (0.1 mol/l citric acid and 0.1 mol/l tri-sodium citrate-2-hydrate) and microwaving at 84°C followed by treatment with pepsin (involving the anti-N-APP antibody, 1:500, MAB348, clone 22C11; Millipore) as described. Before the primary antibody incubation, sections were treated for 1 hour with blocking solution (PBS (pH 7.4) containing 5% normal horse serum, 5% normal goat serum and 4% BSA). Primary antibodies were diluted in PBS (pH 7.4, with 2.5% normal horse serum, 2.5% normal goat serum and 2% BSA added), and sections were then incubated with the primary antibody overnight in a wet histochamber at 4°C under constant agitation. Sections were washed three times for 5 minutes each in PBS(pH 7.4). Secondary antibodies coupled to either Alexa Fluor 488 or Cy3 (diluted 1:1000 and 1:500, respectively, in the same buffer as for primary antibodies; Molecular Probes and Invitrogen, respectively) were added to the sections and incubated for 45 minutes. To reduce auto-fluorescence of intracellular lipofuscin in aged cells, we adapted the protocol described previously [29]. Sections were treated with 0.1% Sudan Black (Carl Roth GmbH, Karlsruhe, Germany) in 70% methanol for 2 minutes in a wet histochamber and briefly washed with PBS before coverslipping. Sections were mounted with aqueous mounting medium containing DAPI (Dako) to visualize cell nuclei. Laser scanning confocal microscopy and digital imaging was performed as described above for the murine immunofluorescence staining.

### Protein extracts and immunoblotting

Mice (Table 1) were decapitated, then the hippocampi and cortices were immediately dissected and sonicated on ice in 5 volumes of RIPA buffer (20 mmol/l Tris, 150 mmol/l saline, 1% Triton X-100, 1% sodium deoxycholate, 0.1% SDS) containing protease (Mini Complete Tablets^TM^™;; Roche, Diagnostics, Basel, Switzerland) and phosphatase inhibitors (Sigma-Aldrich), and separated by centrifugation at 40,000 g at 4°C for 20 minutes. The pellet/membrane fraction was extracted in 20 μl of formic acid and resuspended in 100 μl EBS buffer (50 mmol/l Tris-Cl, pH 8.0, 120 mmol/l saline, 0.5% v/v Nonidet P-40, 5 μg/ml leupeptin, 10 μg/ml aprotinin, 50 μg/ml PMSF, 0.2 mmol/l sodium orthovanadate, 100 mmol/l sodium fluoride). Total protein concentrations of the pellet and supernatant were measured with a spectrophotometer (NanoDrop; Thermo Fisher Scientific Inc. Rockford, IL, USA) or Bradford assay (BioRad laboratories Inc, Hercules, CA, USA), respectively. Protein samples (20 μg) were separated by SDS-PAGE using 10 to 20% Tris-Tricine gels (Invitrogen, Carlsbad, CA, USA), blotted onto 0.1 μm nitrocellulose (Protran; Sigma-Aldrich) (see Table 3 for details). After blotting, the nitrocellulose membranes were dried at room temperature for 20 minutes [30]. Membranes were blocked (Table 3) and incubated overnight at 4°C with the primary antibodies (Table 2). Before and after incubation with the secondary antibodies (horseradish peroxidase-conjugated antibodies; Jackson ImmunoResearch Laboratories), membranes were washed for 1 hour in total (6 × 10 minutes) in Tris-buffered saline with Triton X-100. The signal was visualized by the enhanced chemiluminescence reaction (Amersham Biosciences Europe, Freiburg, Germany) in accordance with the manufacturer’s instructions. Quantitative analyses of the immunoreactive bands were performed on digitalized films using ImageJ software (NIH). Sum pixel brightness values (the integrated density), corrected for non-specific background and equal loading using β-Actin as a control, were averaged per animal and included in the statistical analysis.

**Table 3:** Western blotting parameters

**Antibody**	**Run time, hours/voltage**	**Transfer time hours**	**Blocking reagent/time, hours**	**Secondary antibody incubation, hours**
total Tau/Ab-2	1.5 to 2/125	1.5	10% WBR^1^/1	1.5
PHF-Tau				
pTau^T205^				
APP, C-terminal	2/125 or 2.5/100^3^	1.5	10% FBS^2^/2	1
APP (A4)	3/1253	1.5	10% WBR/1	1.5
β-Actin^4^			10% WBR/1	1

FBS, fetal bovine serum (heat-inactivated); paired helical filament; WBR, Western blocking reagent (Roche Applied Science).

^1^Incubation of membrane in boiling PBS for 5 minutes recommended.

^2^Incubation of membrane in boiling PBS for 5 minutes required.

^3^Western blotting apparatus kept in the water bath at room temperature to prevent overheating.

^4^β-actin was measured for each blot after stripping (2% SDS at 56°C for 30 minutes).

### ELISA

ELISA microtiter plates (catalog number EZBRAIN40/42; Millipore) were used for the quantitative analysis of Aβ_1–40_ and Aβ_1–40/42_. In addition, mouse-specific Aβ and IL-1β ELISA microtiter plates (catalog numbers KMB3481 and KMC0011, respectively; Invitrogen, Camarillo, CA, USA) and a fluorokine multi analyte profiling kit for the detection of IL-1α, IL-6, and tumor necrosis factor α (TNFα; catalog number LUM410; R & D Systems, Minneapolis, MN, USA) were employed to confirm the murine origin of the selected proteins. All assays were performed in accordance with the manufacturer’s protocol. Samples (50 μl) of the brain extracts (supernatant and pellet) or terminally collected blood plasma samples were used and run in duplicate. After 30 minutes incubation in the substrate solution (3,3′,5,5′-tetramethylbenzidine/peroxide mixture), the reaction was stopped and the absorption quantified using a microplate reader (Synergy HT Multi-Mode; BioTek Instruments Inc, Winooski, VT, USA) measuring the difference at 450 nm and 650 nm.

### Statistical analyses

All analyses were performed with SPSS for Windows (version 16; SPSS Schweiz AG, Zug, Switzerland). ANOVA with ‘treatment’ as the main between-subjects factor was performed for the behavioral test, the immunohistochemical and immunoblotting analyses (anti-Aβ, anti-pTau, anti-GFAP, anti-CD68 IR) involving double immune-challenged mice (PP; PolyI:C at GD17 and in adulthood), and their controls for NN (NaCl at GD17 and in adulthood), NP (NaCl at GD17, PolyI:C in adulthood), and PN (PolyI:C at GD17, NaCl in adulthood). Significant main effects were explored further and the corresponding mean values between groups were compared using Fisher's least significant difference (LSD) test. Planned comparisons (NaCl versus PolyI:C, NP versus PP) with Mann–Whitney *U*-tests were used for the single and double immune-challenge experiments involving immunohistochemical, immunoblotting, and ELISA experiments assessing APP/Aβ and pTau levels. Statistical significance was set at *P*<0.05.

## Results

**Figure 1:**
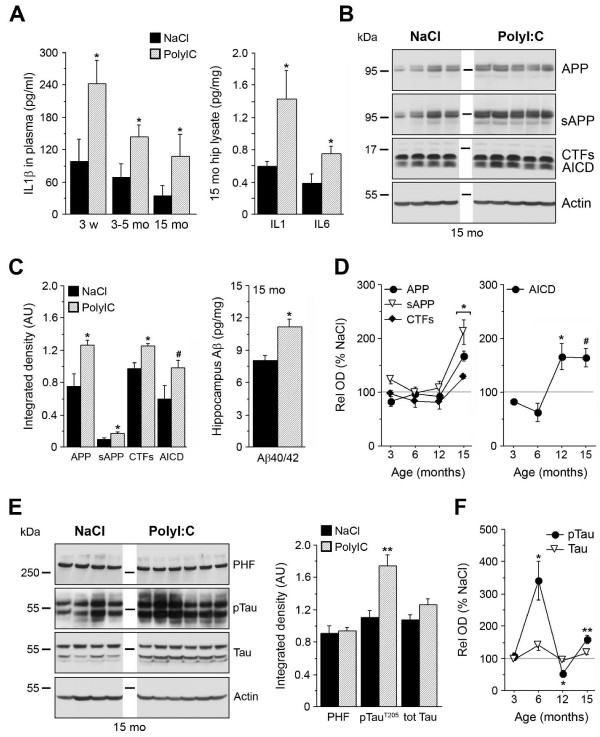
**Prenatal immune challenge results in long-term alterations in interleukin (IL)-1β levels, amyloid precursor protein (APP) processing, and Tau phosphorylation.** (**A**) After prenatal exposure to polyriboinosinic-polyribocytidilic acid (PolyI:C), elevation of the proinflammatory cytokines IL-1β in plasma and brain and IL-6 in brain was observed. (**B,C**) Quantification of APP and its proteolytic fragments in hippocampal lysates of 15 month-old mice. Western blots (WB, left) and ELISA (right) using anti-N- and C-terminal APP, and Aβ_1–40/1–42_ specific antibodies, respectively. WB values represent mean relative optical density normalized to β-actin and expressed in arbitrary units (AU). Lanes represent different animals, and the inset highlights the size markers. (**D**) Overview of longitudinal APP-related biochemical changes (percentage changes relative to saline (NaCl) occurring after prenatal viral-like infection. (**E**-**F**) WB, quantification (15 month-old mice), and longitudinal changes in Tau phosphorylation in mice prenatally exposed to NaCl or PolyI:C, assessed using anti-paired helical filaments (PHFs), anti-pTau^T205^, and anti-total Tau antibodies. Values represent mean ± SEM, n = 4 to 7 mice per treatment and age. **P < 0.01, *P < 0.05, #*P* = 0.08 statistics based on (**A**, **C**, **E**) Mann–Whitney *U*-test or (**D**, **F**) ANOVA/Fisher LSD test.

**Figure 2:**
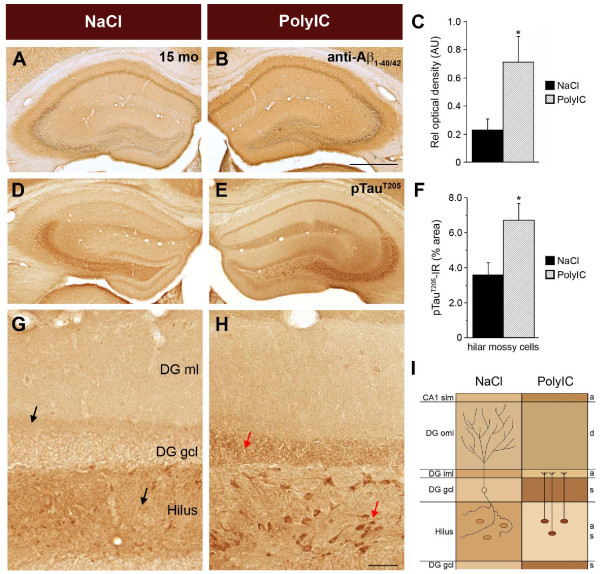
**Long-term changes induced by a prenatal infection include increased anti- amyloid precursor protein (APP)/Aβ immunoreactivity (IR) and somatodendritic accumulation of pTau IR.** Representative images of immunoperoxidase staining using (**A,B**) anti-Aβ_1–40/42_ antibody and (**D,E,G,H**) anti-phosphorylated Tau^205^ taken from the dorsal hippocampus of 15-month-old non-transgenic mice prenatally exposed to saline (NaCl) or polyriboinosinic-polyribocytidilic acid (PolyI:C). Quantitative analyses of the area covered by (**C**) anti-APP*/*Aβ IR or (**F**) anti-pTau IR in hilar mossy cells. Values are given as mean ± SEM, n = 6 per treatment. *P < 0.05; statistical significance based on Mann–Whitney U test. (**D,E**) Translocation of pTau IR from axonal to somatodendritic compartments in the dentate gyrus (DG) and hilus after prenatal infection. (**G-H**) Representative higher-magnification image of the dorsal DG, showing the typical axonal pTau IR in the inner molecular layer of the DG (axonal terminals of contralateral hilar mossy cells, upper arrow) and in the neuropil of the hilus (axonal collaterals of DG granule cells, lower arrow) in NaCI-exposed control subjects. Compared with controls, a pronounced increase in pTau IR in somatic compartments was evident in the PolyI:C-treated offspring, as indicated by the distinct labeling of DG granule cells and hilar mossy cells (red arrows). At the same time, IR in the axonal compartments of the two cell types was strongly reduced. (**I**) Schematic overview of the observed changes within CA1 and DG between treatment groups, emphasizing the change in subcellular distribution of pTau from axonal to somatodendritic compartments. The drawing on the left highlights the dendritic arborization of a DG granule cell in the outer molecular layer, and indicates the axonal collaterals in the hilus. On the right, the projection of the mossy cells to the inner molecular layer of the DG (commissural; that is, targeting the contralateral hemisphere) are shown. Abbreviations: a, axonal projection area; d, dendritic subfield; gcl, granule cell layer; iml, inner molecular layer; oml, outer molecular layer; s, somatic subfield; slm, stratum lacunosum moleculare. Scale bars: B = 500 μm, H = 50 μm.

To elucidate the role of inflammatory processes in the etiology of AD-like pathology in mice, we challenged non-transgenic mouse dams with PolyI:C at GD17, and analyzed the development and progression of AD-like neuropathology in the offspring during adulthood and aging. We first measured the inflammatory cytokine IL-1β, which has been suggested to play a crucial role in the etiology of AD [9-11]. We found that plasma IL-1β levels were significantly increased in animals exposed prenatally to PolyI:C versus saline (NaCl) subjects at the time of weaning, and these levels remained higher throughout aging both in plasma and brain tissue (Figure 1A). In line with the finding that a long-term peripheral infusion of IL-1β induces hippocampal cytokine mRNA expression [31], we also found increased levels of other cytokines, such as IL-1α and IL-6, in the hippocampus of 15 month-old mice exposed to PolyI:C compared with saline controls (Figure 1A; see Additional file 1: Figure S1A-C). In addition, immunohistochemistry using anti-CD68 antibodies, which predominantly labeled activated microglia/macrophages, identified the presence of some microglia cells with altered morphology in the hippocampus CA1 *stratum lacunosum moleculare* (see Additional file 1: Figure S1D-K), indicative of an activated state [32]. Furthermore, prenatal PolyI:C exposure resulted in a significant age-dependent increase in the amount of amyloid precursor protein (APP) and its proteolytic fragments: α/β-secretase-generated ectodomains (soluble (s)APP), C-terminal fragments (CTFs) and their γ-secretase-cleaved APP intracellular domains (AICD), and β/γ-secretase-generated Aβ peptides (Figure 1B-D; see Additional file 2: Figure S2A-C; see Additional file 3: Figure S3A for schematic representation of APP and antibodies used to detect the proteolytic fragments). Whereas the levels of APP, sAPP, and CTFs were found to increase between 12 to 15 months in PolyI:C compared with saline-treated subjects, the levels of AICD, a neuroinflammation-inducing fragment of APP [33], had already reached significantly higher levels by 12 months (Figure 1D). Using densitometric analysis of APP IR, a significant increase in hippocampal APP and its proteolytic fragments was also found in 15 month-old PolyI:C-exposed versus saline-treated mice (Figure 2A-C). Taken together, these results suggest that a late-gestational immune challenge evokes a chronic neuroinflammatory state in the offspring that is accompanied by a significant increase in APP levels and amyloidogenic APP processing in the aged hippocampus.

**Figure 3:**
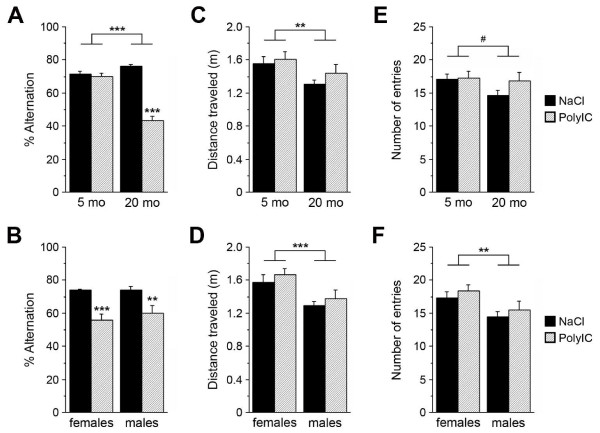
**Age-associated impairments in performance in polyriboinosinic-polyribocytidilic acid (PolyI:C) mice in the Y-maze test.** (**A-B**) ANOVA of the % alternation showed a significant main effect for treatment (*F*_*1,54*_ = 90.1, *P* < 0.001) and age (*F*_*1,54*_ = 39.8, *P* < 0.001), and a treatment × age interaction (*F*_*3,54*_ = 70.6, *P* < 0.001), but no effect for sex (*F*_*1,54*_ = 0.0002, *P* = 0.988). Fisher's LSD post-hoc analysis confirmed the significant impairment in working-memory performance in the 20 month-old PolyI:C-exposed subjects compared with NaCI-treated subjects in (females: *P* < 0.001, males: *P* = 0.003). (**C**-**D**) ANOVA of the total distance travelled identified a significant main effect for age (*F*_1,54_ = 10.0, *P* = 0.003) and sex (*F*_1,54_ = 16.5, *P* < 0.001), but no main effect for treatment (*F*_*1,54*_ = 0.51, *P* = 0.480), indicating that the memory impairment was not confounded by differences in locomotor activity. Post-hoc analysis indicated that males travelled significantly shorter distances than did females (*P* = 0.0003, Fisher's LSD *Post-hoc* analysis). (**E-F**) Similarly, ANOVA of the total number of entries into the Y-maze arms showed no significant main effect for treatment (*F*_*1,54*_ = 0.53, *P* = 0.470), but a main effect for sex (*F*_1,54_ = 11.1, *P* = 0.002), reflecting the lower activity of males relative to females (*P* = 0.002, Fisher's LSD post-hoc analysis*).* Besides the effect for age, which closely approached significance (*F*_1,54_ = 3.8, *P* = 0.056), a significant age × sex interaction emerged (*F*_*3,54*_ = 4.2, *P* = 0.045), indicating the lower activity of old males relative to females. Values are given as mean ± SEM; n = 13 to 18 per age and treatment; ***P < 0.001, **P < 0.01.

Interestingly, levels of phosphorylated (p)Tau^T205^ were also significantly increased in PolyI:C mice relative to controls at 6 and 15 months; however, they were significantly reduced at 12 months of age without significant changes in the levels of PHFs or total Tau (Figure 1E-F; see Additional file 2: Figure S2D-F; see Additional file 3:Figure S3B for schematic representation of Tau and the antibodies used to detect the different phosphorylated epitopes), suggesting dynamic and potentially compensatory changes in the regulation of Tau phosphorylation. Using an optimized antigen-retrieval protocol [26], we were also able to identify, in 15-month-old PolyI:C-treated animals, a striking mis-sorting of pTau from axonal to somatodendritic compartments in hippocampal neurons (Figure 2D-I), a suggested prerequisite for the induction of synaptic dysfunction [34]. In line with these observations, PolyI:C-treated mice showed a significant aging-associated deficit in the Y-maze paradigm, which was not attributable to putative differences in sensory and locomotor activities (Figure 3). Thus, a prenatal immune challenge is sufficient to trigger a series of neuropathologic events that lead to a slow but gradual increase in amyloidogenic APP processing, Tau hyperphosphorylation and mislocalization, and cognitive impairments, potentially representing a state of increased vulnerability of the brain to AD.

**Figure 4:**
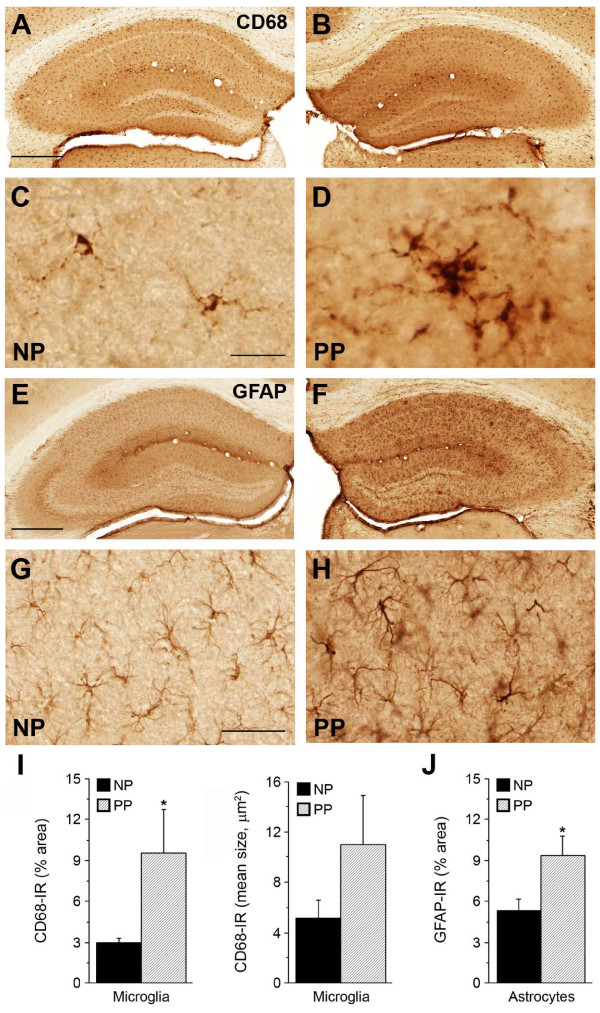
**Microglia activation accompanied by prominent astrogliosis in double immune-challenged non-transgenic mice.** Immunoperoxidase staining of brain sections from 18-month-old NP (NaCl at gestation day (GD)17, PolyI:C at 15 months) and PP (polyriboinosinic-polyribocytidilic acid (PolyI:C) at GD17, PolyI:C at 15 months) mice. Increase in (**A-D**) CD68 and (**E-H**) glial fibrillary acidic protein (GFAP) immunoreactivity (IR) in the PP hippocampus. (**D**) Note the strong hypertrophy of CD68-positive microglia in PP versus NP subjects, indicative of their activated state. Quantitative analysis of hippocampal (**I**) anti-CD68 and (**J**) GFAP IR representing total area and mean size of activated microglia and astrocytes, respectively. Values are given as mean ± SEM, n = 5 per treatment. *P < 0.05; statistical significance based on Mann–Whitney U test. Scale bars: (**A**,**E**) = 500 μm; (**C**) = 20 μm, (**G**) = 50 μm.

Based on these findings, we hypothesized that a second systemic immune challenge in adulthood might exacerbate the development of Aβ and Tau neuropathology in non-transgenic mice. To test this, mice prenatally exposed to PolyI:C (P) and their saline (N) controls were challenged during aging (between 9 and 15 months) with a second PolyI:C injection, or treated with saline, yielding four treatment groups for comparison (NN, NP, PN, PP; Table 1). Three months after the immune challenge, biochemical and/or immunohistochemical analyses were performed to examine the effect of the second exposure on AD-like neuropathology. Having seen alterations in microglial morphology/activity in the prenatally challenged cohort, we first analyzed potential changes in CD68. Immunohistochemical analysis showed a significant increase in CD68 IR in double immune-challenged mice compared with controls (Figure 4A-D,I; see Additional file 4: Figure S4). In addition to this overall increase in CD68 IR, there were widespread changes in both size and morphology of microglia, specifically in the hippocampus, indicating the activated state [32] (Figure 4D). The microglial changes in double immune-challenged mice were accompanied by a massive hippocampal astrogliosis (PP versus NP; Figure 4E-H,J; see Additional file 5: Figure S5). Based on these results, we suggest that prenatal immune challenge induces a subacute but persistent activation of microglia [35], which is probably responsible for the rise in inflammatory cytokines during adulthood, and may even induce a slow but gradual deterioration of the brain’s innate immune system during aging [36]. A second immune challenge that is accompanied by pronounced astrogliosis is expected to accelerate this process, ultimately resulting in dysregulation of brain metabolism and strongly affecting neuronal health [37].

**Figure 5:**
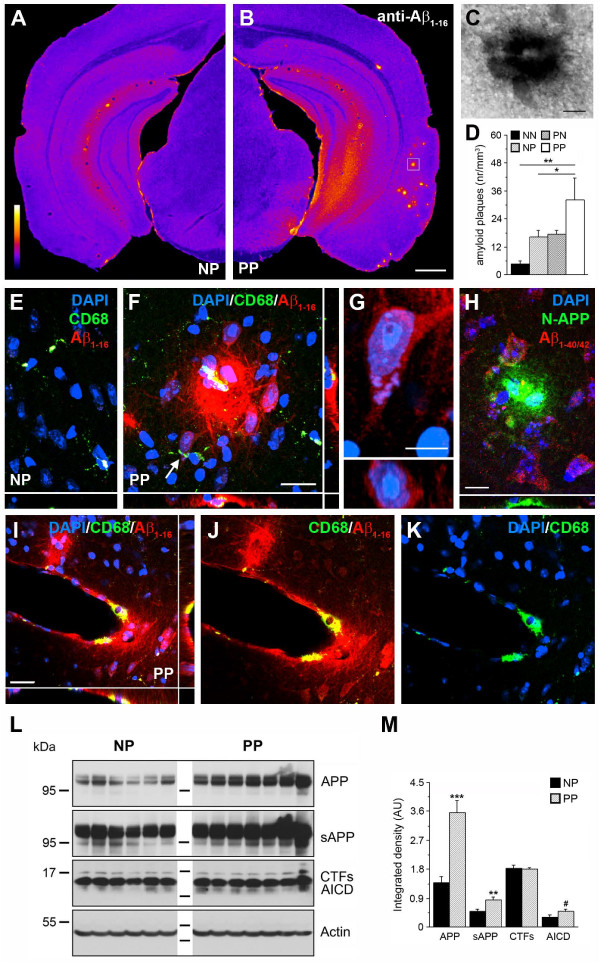
**An adult immune challenge induces amyloid precursor protein (APP) accumulation in non-transgenic mice prenatally exposed to polyriboinosinic-polyribocytidilic acid (PolyI:C).** (**A,B**) Coronal brain sections of 15-month-old single (NP, NaCl at gestation day (GD)17, PolyI:C at 12 months) and double (PP, PolyI:C at both GD17 and 12 months) immune-challenged mice processed for anti-Aβ_1–16_ immunoperoxidase staining. Images are color-coded for visual display. (**C**) Enlarged view (box in **B**) of a representative precursor plaque. (**D**) Quantitative analysis of the numerical plaques density in all four treatment groups. Values are given as mean ± SEM, n = 6 to 7 per treatment. *P < 0.05, **P < 0.01; statistical significance based on ANOVA and Fisher LSD post-hoc analysis. (**E**-**F**) Confocal images captured in the entorhinal cortex of NP and PP brain sections processed for triple immunofluorescence staining. Activated microglia (CD68, green) were found in close vicinity to the plaques (anti-Aβ_1–16_) in PP mice. (**G**) Enlarged neuron in the entorhinal cortex (arrow in **F**) counterstained with DAPI (blue) displaying intracellular anti-Aβ_1–16_ IR (red). Bottom image shows confocal view along the xz axis. (**H**) Anti-N-terminal APP antibody (green) revealed strong accumulation of APP ectodomains in extracellular deposits located in the entorhinal cortex (PP mouse), whereas anti-Aβ_1–40/42_ (red) IR was seen only in surrounding neurons. (**I**-**K**) Pronounced accumulations of APP deposits (anti-Aβ_1–16,_ red) in the cerebral vasculature in PP mice were associated with microglia (anti-CD68 antibody, green). Blue channel shows the DAPI nuclear counterstaining. (**L**) Western blots of hippocampal lysates obtained from 12-month-old NP and PP mice using anti-N-terminal and anti-C-terminal APP antibodies. (**M**) Quantitative analysis of the immunoreactive signals. Values represent mean relative optical density normalized to β-Actin and expressed in arbitrary units (AU) (mean ± SEM, n = 6 to 7). ***P < 0.001, **P < 0.01, *P < 0.05, #*P* = 0.08; statistics is based on Mann–Whitney U test. Scale bars: (**B**) = 500 μm, (**C**,**F**,**I**) = 20 μm, (**G**) = 5 μm, (**H**) = 10 μm.

To test whether the pathological changes seen in glial cells of double immune-challenged mice coincided with an aggravation of AD-like neuropathology, we combined immunohistochemistry and biochemistry to assess amyloidogenic APP processing. Using optimized anti-Aβ immunoperoxidase staining [26], we found a pronounced amyloid-like plaque deposition in double immune-challenged mice compared with controls (Figure 5A-G). The pathology was most prominent in the anterior piriform and lateral entorhinal cortices and their axonal projection areas (Figure 5A-B; see Additional file 6: Figure S6), which are among the first areas affected in AD [38]. In line with observations from human patients with AD [39,40], some of the plaques were associated with the cerebral vasculature (Figure 5I-K) and accompanied by activated microglia (Figure 5F,I). To investigate the composition of these plaques, double-immunofluorescence staining was performed using the N-terminal specific anti-APP and anti-Aβ_1–40/42_ antibodies. This showed a pronounced accumulation of APP and its fragments containing the N-terminal domain in the deposits (Figure 5H), whereas Aβ/CTFs detected with anti-Aβ_1–40/42_ antibodies were only enriched in cells closely associated with the plaques (Figure 5H). Biochemical evaluations confirmed the significant increase in APP, sAPP, and AICD levels after a second immune challenge (Figure 5L-M). However, neither biochemistry nor immunohistochemistry investigations showed any evidence of a significant accumulation of mouse Aβ in these APP depositions, which might be because of the different aggregation properties of rodent compared with human Aβ peptides, or might be linked to the premature age and disease stage of these animals.

**Figure 6:**
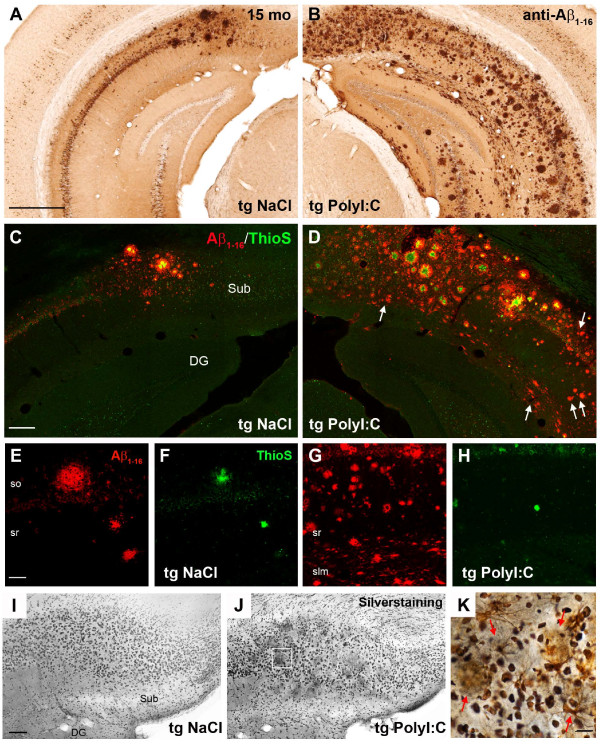
**Single immune challenge in adults induces a dramatic increase in Aβ plaque burden and prominent neurodegeneration in 3xTg-AD mice.** Representative images of (**A**-**B**) immunoperoxidase labeling, (**C**-**H**) immunofluorescence, and (**I**-**K**) silver staining of coronal brain sections of 15-month-old 3xTg-AD mice (Tg) exposed to polyriboinosinic-polyribocytidilic acid (PolyI:C) or NaCI (NaCl) at 4 months of age. (**A**-**B**) The typical anti-Aβ_1–16_ IR seen in the NaCI-treated mice was significantly aggravated after PolyI:C exposure. (**C**-**D**) Counterstaining revealed newly formed Aβ plaques in subiculum and CA1 (arrows) that were thioflavinS (ThioS)-negative, suggesting the non-fibrillary nature of the immune challenge-induced plaques. (**E**-**H**) Representative images of the ventral CA1 area of (**E**, **F**) NaCI-treated and (**G**,**H**) PolyI:C-treated transgenic animals, highlighting the striking increase in ThioS-negative Aβ plaques after a single immune challenge. (**I**-**K**) The increased plaque deposition in PolyI:C-treated 3xTg-AD mice was accompanied by distinct neurodegeneration. Note the dark precipitates in the vicinity of large plaques in PolyI:C treated mice. (**J**,**K**) Higher-magnification color image acquired in the marked area in (**J**) showing the dense silver precipitates, indicative of dystrophic neurites (red arrows), surrounding Aβ plaques (gray/yellow). Abbreviations: so, stratum oriens; sr, stratum radiatum; slm, stratum lacunosum moleculare; Sub, subiculum; DG, dentate gyrus. (**A**) = 500 μm; (**C**) = 150 μm; (**E**; **K**) = 50 μm; (**I**) = 100 μm.

**Figure 7:**
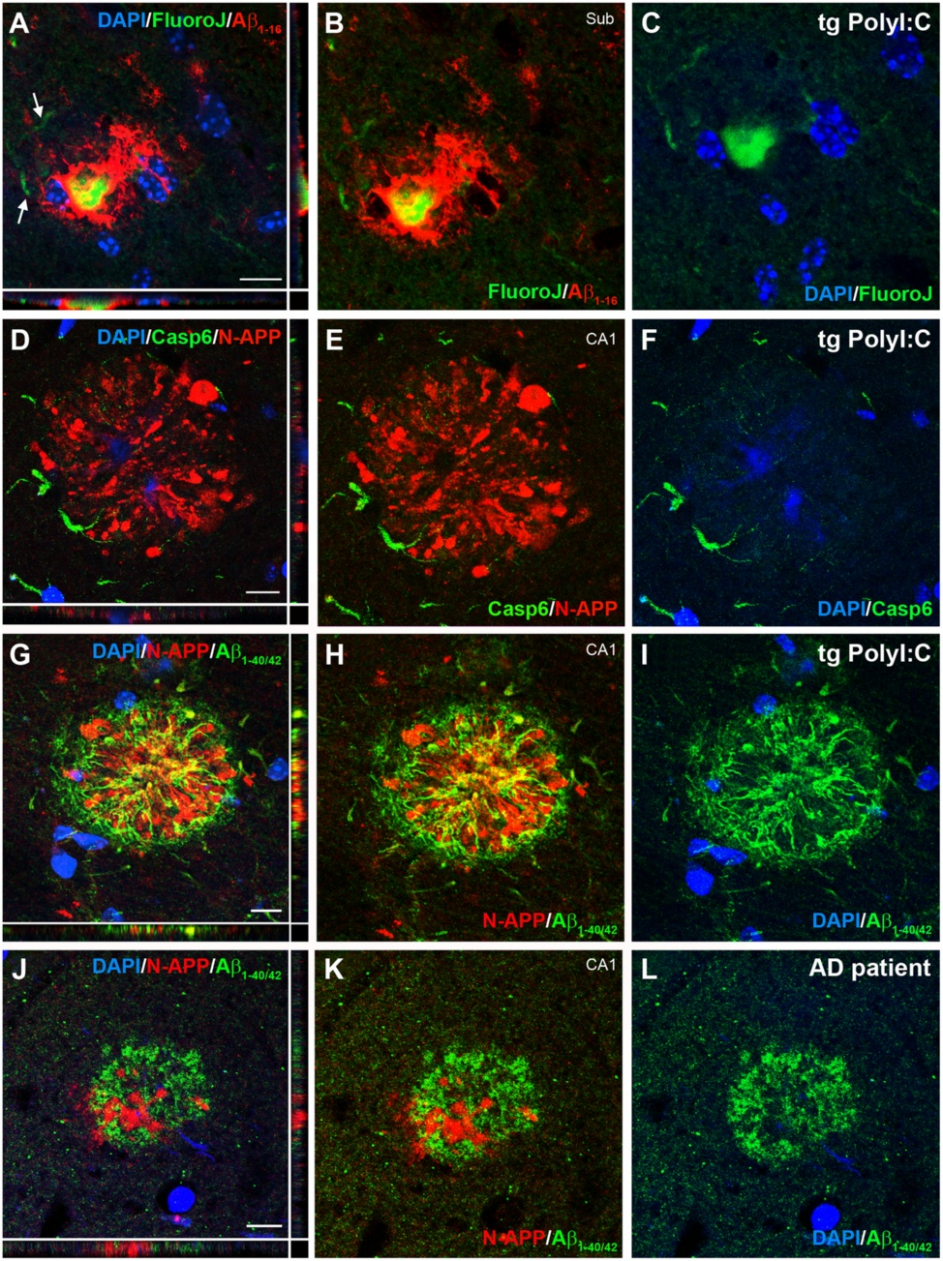
**Characterization of the inflammation-induced Aβ plaques in 3xTg-AD mice.** (**A**-**C**) Immunofluorescence staining using anti-Aβ_1–16_ antibody (red) counterstained with Fluoro-Jade (green) and DAPI (blue) revealed, in addition to the dystrophic neurites (arrows), the presence of degenerating neurons in the core of amyloid deposits in immune-challenged transgenic mice. (**D**-**F**) Double-immunofluorescence staining using anti-N-APP (red) and anti-activated caspase-6 (green) confirmed the presence of dystrophic neurites within and around the amyloid deposits. (**G**-**I**) Transgene-driven human Aβ aggregates (green, anti-Aβ_1–40/42_) around the APP/soluble (s)APP (red, anti-N-APP) accumulations in PolyI:C-treated 3xTg-AD mice, in striking similarity to the phenotype seen in human patients with AD (**J**-**L**). Note the close association but no colocalization between APP ectodomains and C-terminal, Aβ-containing fragments (**G**-**L**). Scale bars = 10 μm.

To test whether inflammation-induced accumulation of APP and its fragments may represent a seeding point for senile, human-like Aβ deposits, we used transgenic mice (3xTg-AD), an established mouse model of AD [23], and challenged them with PolyI:C at the pre-plaque stage of 4 months [23]. Immunohistochemical analysis at 15 months showed a dramatic increase in Aβ plaque deposition in the entire hippocampus of PolyI:C-treated compared with saline-treated mice (Figure 6A-B; see Additional file 7: Figures S7 and Additional file 8: Figure S8). Except for the large subicular plaques, most of the deposits were thioflavinS-negative, indicating that the inflammation-induced plaques were not fibrillary in nature (Figure 6C-H). However, most of these plaques contained Fluoro-Jade-positive cores (Figure 7A-C) and were surrounded by activated caspase 6-positive (Figure 7D-F) and silver-positive dystrophic neurites (Figure 6I-K), indicating degenerative processes accompanying amyloid deposition in immune-challenged 3xTg-AD mice. Importantly, inflammation-induced plaques contained significant amounts of proteolytic APP fragments (Figure 7D,G), as seen in double immune-challenged WT mice (Figure 5H), and were surrounded by Aβ peptides (Figure 7G-I) that had a striking similarity to the morphology of senile Aβ plaques seen in human patients with AD (Figure 7J-L; see Additional file 9: Figure S9). These results suggest that the inflammation-induced APP depositions represent a seed for amyloid plaques in both rodents and humans.

**Figure 8:**
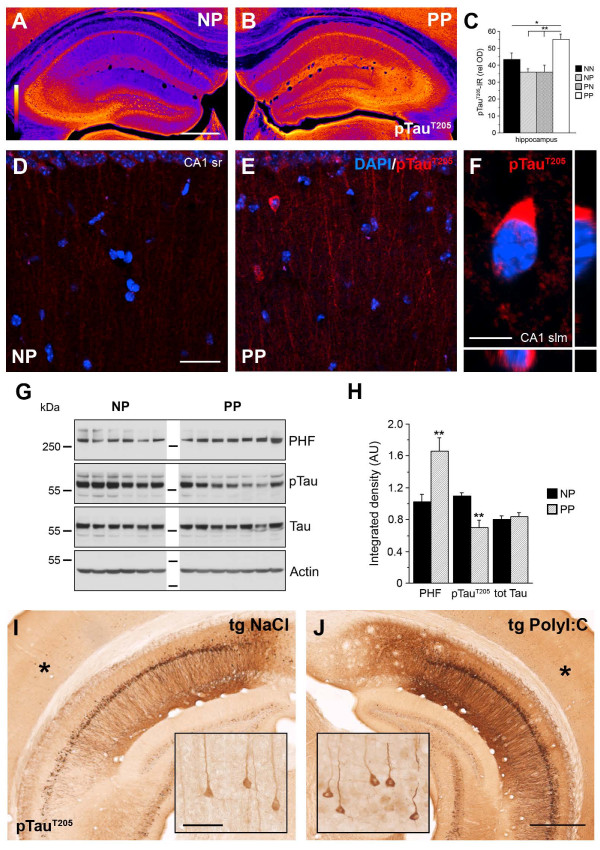
**Aggravated Tau pathology after systemic infection in adult mice.** Representative images of coronal brain sections obtained from (**AD**) NP (NaCl at gestation day (GD)17, PolyI:C at 12 months) and (**BEF**) PP mice (PolyI:C at GD17 and at 12 months), processed for (**A,B**) immunoperoxidase and (**DF**) immunofluorescence staining using anti**-**phosphorylated Tau^T205^ antibodies. (**AB**) Images are color-coded for visual display, with white/yellow indicating highest and blue/purple lowest staining intensity. (**C**) Quantitative analysis of the optical density (OD) of pTau IR in the hippocampus of all treatment groups. Values represent mean OD values corrected for non-specific background labeling (mean ± SEM), n = 6 to 7. **P < 0.01, *P < 0.05; statistical significance based on ANOVA/Fisher’s LSD post-hoc analysis. (**DF**) Increase in pTau IR in CA1 is accompanied by mis-sorting into dendrites and intraneuronal aggregation in non-transgenic PP mice. (**F**) Enlarged view of the CA1 stratum lacunosum moleculare subfield showing the typical somatic pTau aggregation. (**G**) Biochemical analysis of Tau phosphorylation using anti-paired helical filaments (PHFs), anti-pTau^T205^, and anti-total Tau antibodies. Lanes represent individual mice. (**H**) Quantitative analysis of the immunoreactive signals. Values represent mean relative optical density normalized to β-Actin and expressed in arbitrary units (AU) (mean ± SEM, n = 6 to 7). **P < 0.01; Mann–Whitney U test. Please note that the pTau^T205^antibody was used for (**AB**) immunohistochemistry (IHC) and (**G**) immunoblotting (IB) experiments. Because this antibody can crossreact with Ser199 in mouse Tau, an epitope that can be detected with the AT100 (PHF) antibody [63], the increase of pTau IR in (**AB**) is potentially due to an increase in aggregated pTau in sections. (**IJ**) Anti-pTau^T205^ IR in the ventral hippocampus of 15-month-old 3xTg-AD mice injected with NaCI or PolyI:C at 4 months. Insets show higher magnifications of the cortical areas (asterisk) with low transgene expression, and indicate formation of distinct neurofibrillary tangle-like structures after a single systemic infection in transgenic AD mice. Scale bars: (**A**) = 500 μm, (**D**) = 30 μm, (**F**) = 5 μm, (**I,** inset) = 50 μm, (**J**) = 500 μm.

Apart from the amyloid pathology, we also investigated the effect of a second immune challenge on Tau pathology. In double immune-challenged WT mice (PP), immunohistochemical staining showed a significant increase in pTau IR in all subfields of the hippocampus (Figure 8A-C), accompanied by widespread mis-sorting and aggregation of phosphorylated Tau in neuronal somata, compared with control mice (Figure 8D-F). We also detected a significant increase in the level of PHF-Tau, whereas soluble pTau^T205^ levels were significantly reduced (Figure 8G-H), indicating abnormal aggregation of pTau after the double immune challenge. However, we did not observe neurofibrillary tangles (NFTs) in PP mice. Analysis of the Tau phosphorylation levels in immune-challenged 3xTg-AD mice showed that, apart from a strongly increased pTau IR level in the hippocampal formation (Figure 8I-J; see Additional file 8: Figures S8 and Additional file 10: Figure S10), a single PolyI:C injection during pre-plaque stage was sufficient to induce tangle-like structures in neuronal somata outside the high *tau* transgene expression sites, including the somatosensory cortex (Figure 8I-J, inset). It is conceivable, therefore, that sustained activation of microglia, Aβ accumulation, and/or chronic neuroinflammation is required to induce the development of NFTs in non-transgenic mice [41].

Taken together, the results presented here demonstrate that systemic immune challenges not only represent a crucial trigger but also a potent driver of AD-like neuropathology in both environmentally (PolyI:C exposure at GD17) and genetically (3xTg-AD) predisposed animals, in agreement with the notion that peripheral infections and inflammation in patients with AD are associated with more rapid cognitive decline and exacerbation of their symptoms [42].

## Discussion

In this study, we provide the first evidence that a systemic immune challenge during a late-gestational time window (GD17) predisposes WT mouse offspring to pathological brain aging and cognitive decline. A second immune challenge aggravates this phenotype and is sufficient to precipitate significant and widespread amyloid-associated and pTau-associated neuropathology in the aged offspring. By administering a single systemic infection to genetically predisposed transgenic mice (3xTg-AD) overexpressing the human variants of AD-relevant genes [23], we confirmed that the AD-like neuropathological hallmarks seen in double immune-challenged WT mice represent an early AD precursor stage, which is also seen in human patients with AD. Thus, the PolyI:C model offers a unique opportunity to identify molecular targets that are strongly affected by chronic inflammatory conditions and involved in the initiation of the typical neuropathology characteristic of sporadic AD.

Our experimental design builds on our previous studies, in which we showed that middle (GD9) and late (GD17) gestational periods correspond to two windows of enhanced vulnerability to inflammatory cytokines [19]. Whereas mid-gestational immune challenges resulted in neurochemical and behavioral impairments that were releveant for schizophrenia [20], late-gestational PolyI:C exposure has a detrimental and long-term effect on cognitive performance during adulthood and aging [19], suggesting a causal link between disturbances of late embryonic development and risk of AD-like neuropathology [22].

In this study, we extended our previous findings that PolyI:C injection at GD17 induced a significant elevation of maternal and fetal cytokines at both protein and mRNA levels [19], by showing that this insult also has long-term consequences on basal cytokine levels in adulthood. We detected significantly increased levels of both circulating and brain IL-1β in PolyI:C-treated versus saline-treated mice throughout aging. It is conceivable that these changes are linked to the detrimental effect of the prenatal immune challenge on the developing microglia, the macrophage-derived immune cells of the brain that start to colonize the brain during late-gestational stages [43,44], potentially affecting their proliferative and also phagocytotic capacity during adulthood and aging. In addition, the immune challenge may prime or activate microglia, thereby creating an innate immune memory, allowing a faster and exaggerated response upon further immune challenges and exposures to adverse stimuli [35]. Indeed, in double immune-challenged mice we found a pronounced increase in activated microglia in the hippocampus before the accumulation of extracellular amyloid depositions. Furthermore, chronic elevation of several cytokines after a late-gestational immune challenge may also have a detrimental effect not only on neurons but also on microglia, by promoting cytokine-mediated release of reactive oxygen species [45]. In combination with additional immune challenges in adulthood, these pathophysiological changes may detoriate, resulting in loss of the neuroprotective functions of microglia and even microglial degeneration, which in turn may contribute to the spread of neuropathology in AD. Experimental evidence for this hypothesis is provided by *in vitro* findings showing that repetitive stimulation of microglia by mitogens induces telomere shortening and replicative senescence [46], and that interferon-γ exposure is sufficient to trigger activation-induced microglia cell death [47]. Hence, further morphological and biochemical investigations will be required to determine whether microglia in the aged immune-challenged WT mice may also share characteristics of fragmentation and degeneration, a distinct neuropathologic feature preceding the spread of tau and neuritic plaque pathology in patients with AD [48].

In this study, we also found that chronic rises in inflammatory cytokines are accompanied by significant rises in APP and its proteolytic fragments, and an increase in Tau phosphorylation and somatodendritic mislocalization in aged animals. These observations are in agreement with data showing that ectopic IL-1 application increases APP production and its processing [10,49,50], and increases Tau hyperphosphorylation [51]. In addition, we found that an increase in C-terminal APP fragments, including AICD, which potentially contributes to the neuroinflammation response [33], was one of the first significant changes discovered in the course of aging of prenatally challenged animals. It is also plausible, however, that an early life insult may result in long-lasting epigenetic changes in the promoter region of AD risk genes, as previously proposed [52], providing an alternative explanation for the chronic inflammatory state occurring after prenatal immune challenge.

Based on recent findings showing that systemic inflammation exacerbates the course of the disease both in patients with AD [42] and in rodent models of the disease [53], we tested the effect of the second immune challenge in adulthood on AD-relevant neuropathology. Analysis of these double immune-challenged mice showed a strong aggravation of the phenotype that was present after a single prenatal infection. Moreover, we detected the appearance of extracellular APP/Aβ-positive deposits in the entorhinal and piriform cortex and the formation of intracellular Tau aggregates, selectively in the hippocampal formation. Hence, dysfunction of the brain’s immune system, triggered through systemic infections, may play a key role in initiation of AD pathogenesis, in line with previously proposed hypotheses [37,54].

The existence of APP/Aβ-positive deposits in double immune-challenged non-transgenic mice, in brain areas which are among the first affected in humans [38] indicated that better characterization of these amyloid-like plaques is needed. Using N-terminal specific anti-APP and anti-Aβ_1–40/42_ antibodies, we showed that, whereas the deposits consisted of APP and N-terminus-containing APP fragments, Aβ/CTFs were enriched only in cells closely associated with plaques. The absence of significant accumulations of mouse Aβ peptides in these APP deposits might be due to the different aggregation properties of the rodent compared with the human form [55], or might be linked to the premature age and disease stage in these animals (15 months). It will therefore be important to determine the precise identity of the different APP fragments produced in aged single and double immune-challenged mice.

**Figure 9:**
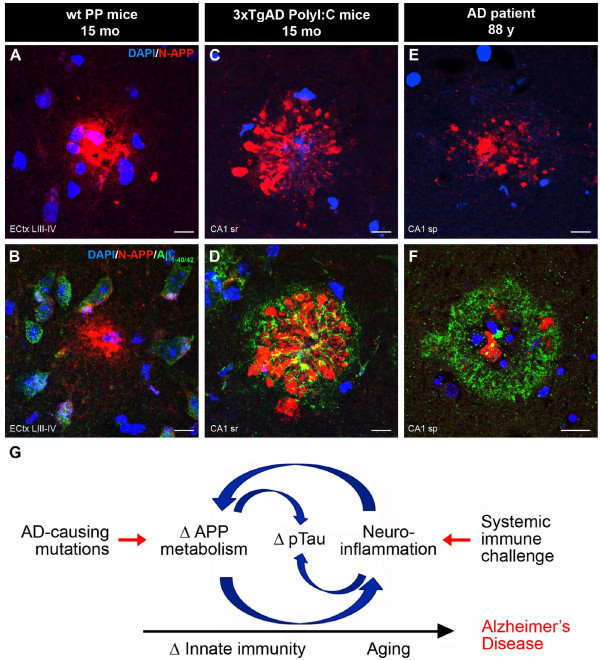
**Inflammation-induced amyloid precursor protein (APP) accumulations represent an early neuropathologic feature in the pathogenesis of Alzheimer’s disease** (**AD).** Extracellular APP or soluble (s)APP-containing amyloid plaques in rodents and humans share striking morphological similarities. Tissue sections obtained from (**A**-**B**) 15-month-old double immune-challenged (polyriboinosinic-polyribocytidilic acid (PolyI:C) at GD17, PolyI:C at 12 months) wild-type (WT) mice, (**C**-**D**) 1-month-old 3xTg**-**AD mice exposed to PolyI:C at 4 months, and (**E**-**F**) an 88-year-old patient with AD. Images were acquired with the same confocal settings in representative areas within the hippocampal formation: layer III-IV in the lateral entorhinal cortex (ECtx, PP), CA1 stratum radiatum (sr, 3xTg**-**AD mice), and CA1 stratum pyramidale (sp, human subject). Antibody used: anti-N-APP (red) and anti-Aβ_1–40/42_ (green). (**G**) Model proposing an AD mutation-independent role of systemic immune challenges, persistent neuroinflammation, and deterioration of innate immunity in the etiology of the age-associated, sporadic form of AD. Scale bars: (**A**-**E**) = 10 μm; (**F**) = 20 μm.

In the current study, we tested the possibility that the inflammation-induced accumulation of APP and its proteolytic fragments may represent a seeding point for fibrillary Aβ deposits. For that we used the 3xTg-AD mice [23] and challenged them with PolyI:C at the pre-plaque stage. Subsequent analysis at 15 months showed a dramatic increase in Aβ plaque deposition in the entire hippocampus of the PolyI:C-treated mice. These data are in line with recent findings of accelerated Aβ pathology in transgenic AD mice after induction of osteoarthritis, which is accompanied by significant upregulation of inflammation-related genes and astrocyte and microglial activation in the brain [56]. Importantly, inflammation-induced plaques contained significant amounts of proteolytic APP fragments, as seen in double immune-challenged WT mice (Figure 9A-D). These APP-derived aggregates were surrounded by human Aβ peptides, showing a striking similarity to the morphology of Aβ plaques seen in human patients with AD (Figure 9E-F).

Importantly, other laboratories have also reported rodent APP in the core of Aβ deposits in variuos transgenic AD mouse strains [55,57]. Hence, we conclude that accumulation of APP and its N-terminus-containing fragments precedes the formation of senile plaques, representing a crucial and conserved precursor stage of this typical neuropathologic hallmark.

In agreement with recent experimental evidence showing that hyperphosphorylated, non-fibrillary Tau may have a key role in eliciting behavioral impairments *in vivo*[34,58], we were able to show in this study that the Tau hyperphosphorylation and mislocalization into somatodendritic compartments is accompanied by severe deficit in prenatally challenged mice compared with saline-treated controls. However, it would be interesting to confirm if a second immune challenge during aging results in progressive decline of cognitive functions, as indicated by the recent findings in patients with AD, providing evidence that acute and chronic systemic inflammation is associated with an increase in cognitive decline [42]. Although we did not observe the formation of NFTs in immune-challenged mice, a second immune challenge in our model elicited the intraneuronal aggregation of Tau, probably involving the formation of PHFs. Further studies will be required to characterize the nature of these and potentially other phosphorylated epitopes affected by the chronic inflammatory state. We have recently shown that NFT-like structures can form in the brain of AD mice lacking *tau* transgene expression, by genetic reduction of the *reelin* gene [27]. Interestingly, formation of these NFT-like structures was associated with a high Aβ plaque burden, and prominent neuroinflammation and neurodegeneration [27]. Hence, it is reasonable to suggest that the formation of PHFs we saw in double immune-challenged WT mice would eventually develop into NFTs at older age and later stages of the disease.

In summary, and based on the results presented and discussed here, we propose a model (Figure 9G) in which the mendelian mutations underlying familial AD cause profound changes in APP metabolism, inducing a neuroinflammatory response capable of driving and aggravating AD neuropathology during aging. Repeated systemic immune challenges, by contrast, induce chronic neuroinflammation and may accelerate senescence of microglia cells. Reduction in their neuroprotective function is expected to impair both APP and pTau homeostasis. This in turn could trigger a vicious cycle that will lead, over the course of aging, to the formation of senile Aβ plaques and NFTs, induce degeneration, and ultimately result in clinical dementia. We suggest, therefore, that systemic infections and persistent neuroinflammatory conditions represent major risk factors for the development of sporadic AD in older persons. Importantly, our model complements the long-standing amyloid hypothesis of AD [59,60], and is in accordance with the recent proposal to revise the current view on the etiology of sporadic AD [61].

## Conclusion

We provide experimental evidence for a causative and exacerbating role of systemic immune challenges on the development of AD-like neuropathology *in vivo*, thereby supporting recent genome-wide association studies [4], retrospective epidemiological human studies [5,6,12], and the long-standing infection/neuroinflammation-based AD hypotheses [11,22,33,61]. Furthermore, our data support the use of anti-inflammatory drugs [12] in the treatment of patients during the initial asymptomatic stages of AD [16], and confirm the importance of identifying early molecular markers of the disease. Finally, with the novel findings of focal accumulations of APP and its proteolytic fragments preceding senile plaque deposition, we offer a very suitable mouse model to elucidate the molecular mechanisms underlying the earliest stages of the typical neuropathologic changes characteristic of sporadic AD.

## Abbreviations

BSA: Bovine serum albumin; DAPI: 4',6-diamidino-2-phenylindole; PBS: Phosphate-buffered saline.

## Competing interests

The authors also declare that they have no competing financial or personal interests, and that none of the author's institutions have contracts relating to this research through which it may stand to gain financially now or in the future.

## Authors’ contributions

DK established the protocols and carried out the biochemical studies, participated in the quantitative analyses, and drafted and co-wrote the manuscript. AmM performed the biochemical analysis involving the time course following prenatal infection. JD, CI, AbM performed the immunohistochemistry and image analyses of the rodent brain tissue. TN carried out all the human post-mortem immunofluorescence staining and performed the quantitative analyses. PV and MH performed biochemical and immunohistochemical analyses involving rodent tissue. SP established the FluoroJ assay and participated in the immunohistochemical experiments. CS and CR carried out the immunoassays. UM carried out the behavioral tests. IK designed the study, performed the statistical analysis, coordinated the experiments, and co-wrote the manuscript. All authors read and approved the final manuscript.

## Supplementary Material

Additional file 1 **Figure 1.** Chronic increase in proinflammatory cytokines following a prenatal immune challenge in the hippocampus but not neocortex. (**A-C**) ELISA of neocortical (Ctx) and hippocampal (Hip) brain lysates obtained from 15-month-old polyriboinosinic-polyribocytidilic acid (PolyI:C)-treated and NaCI-treated mice (n = 4–7 per treatment group). Values represent mean ± SEM. *P < 0.05, Mann–Whitney *U*-test. (**D-K**) Immunohistochemical staining using anti-CD68 antibodies to visualize microglia in the hippocampus. Representative images are taken at different magnification from the dorsal CA1 of 15-month-old mice treated with (**D,F,H**) NaCI and (**E,G,I**) PolyI:C. Higher-magnification images show the activated stage (arrow) in microglia in the CA1 stratum lacunosum moleculare (slm) in 15-month-old mice (**H**) treated with PolyI:C compared with (**I**) control mice. (**J,K**) Anti-CD68 immunoreactivity (IR) in young (8 weeks) and old (15 months) naive wild-type (WT) mice. Whereas very small somata and thin processes dominated in young mice (**J)**, microglia in old naive mice covered larger areas (**K**), very similar to the situation in old NaCI-exposed mice. Scale bars: (**D**) = 500 μm; (**F**) = 100 μm; (**H, J**) = 20 μm. Click here for file

Additional file 2 **Figure 2.** Changes in amyloid precursor protein (APP) processing and Tau phosphorylation across aging after a single prenatal immune challenge in non-transgenic mice. Longitudinal study involving murine hippocampal brain lysates obtained from **(A,D)** 3-month-old, (**B,E**) 6-month-old, and (**C,F**) 2-month-old non-transgenic mice exposed *in utero* at gestation day (GD)17 to either polyriboinosinic-polyribocytidilic acid (PolyI:C) or NaCI. The latter cohort also received a single NaCI injection at 9 months, and constituted the control group for the double immune-challenged mice. Western blots were performed using the following antibodies: (**A-C**) mouse anti-APP A4 (clone 22 C11, recognizing full-length APP, and soluble α- or β-secretase-cleaved APP ectodomains (sAPP); rabbit anti-C-terminal APP (A8717, recognizing α- or β-secretase-generated C-terminal fragments (CTFs) and γ-secretase-cleaved APP intracellular domains (AICD)); and mouse anti-β-Actin (clone 4). (**D-F**) Western blots using mouse anti-paired helical filaments (PHFs, clone AT100), rabbit anti-phosphorylated Tau^205^, and mouse anti-total Tau (Tau-5) antibodies. Quantitative analysis involved the measurement of the integrated pixel brightness of the immunoreactive bands, corrected for non-specific background and equal loading using β-actin as control. Please note that the pTau blot in E has been overexposed for visual display. Western blot lanes show the different subjects. Values represent mean relative, β-actin-corrected optical density expressed in arbitrary units (AU) (mean ± SEM, n = 4 to 6 per treatment and age). *P < 0.05, **P < 0.01, # *P* = 0.053, Mann–Whitney *U*-test. Click here for file

Additional file 3 **Figure 3.** (**A**) Schematic representation of the domain structure of the amyloid precursor protein (short form, APP695), indicatingα-, β- and γ-secretase cleavage sites and antibody binding sites. Note that antibody Aβ_1–16_ (6E10) recognizes both Aβ/C-terminal fragments (CTFs) as well as full-length APP on western blots and tissue sections (see Figure 5). By contrast, antibody Aβ_1–40/42_ has very low affinity for full-length APP but strongly binds to Aβ/CTF fragments. (**B**) Schematic representation of the human Tau protein (441 amino acid, longest isoform). Alternative splicing of mRNA from a single gene located on the long arm of chromosome 17 yields six different isoforms that are expressed in the adult human brain [62]. They differ by the presence of one to two amino-terminal inserts (blue) and three to four tandem repeats (purple) in the carboxyterminal region. The sequence common to all known isoforms is shown in gray. The phosphorylation-dependent anti-Tau antibodies used are listed (including their alternative names) along with the respective phosphorylation-site and flanking sequences. As can be seen from the alignment of the tau sequences in five species, there is a high level of homology between mouse and human tau. Note that pTau^T205^ antibody also crossreacts with Ser199 in mouse Tau, an epitope that can also be detected with the AT100 (PHF) antibody [63] Abbreviations:CTF, C-terminal fragment; AICD, APP intracellular domain; PM, plasma membrane; sAPPα/β,soluble α- or β-secretase-cleaved. Click here for file

Additional file 4 **Figure 4.** Microglia responses in double immune-challenged non-transgenic mice. **(A-D)** Low-magnification images of immunoperoxidase staining using anti-CD68 antibody (MCA 341R) of coronal brain sections obtained from 18-month-old control mice (NN; NaCI at gestational day (GD) and 15 months), and prenatal (PN; polyriboinosinic-polyribocytidilic acid (PolyI:C) at GD17 and NaCI at 15 months), adult (NP; NaCI at GD17 and PolyI:C at 15 months), and double (PP, PolyI:C at GD17 and 15 months) immune-challenged mice. **(D)** A pronounced increase in the density of activated CD68-positive microglia with hypertrophied and ameboid morphology was found throughout the hippocampal formation of double immune-challenged mice compared with control subjects. Note that also immune challenge **(B)** prenatally and **(C)** in adulthood alone had a stimulating effect on microglia OD. **(E-F)** Quantitative analysis of the immunoreactive signals labeled with antibodies against rat CD68. **(E)** ANOVA yielded a main effect of treatment for the quantification of percentage area covered by anti-CD68 immunoreactivity (IR) in the hippocampus (*F*_3_,_16_ = 4.4, *P* = 0.019) with significant differences between NN versus PP (*P* = 0.004), NP versus PP (*P* = 0.015), and PN versus PP (*P* = 0.026). **(F)** A significant main effect also emerged for the mean area (*F*_3_,_16_ = 3.2, *P* = 0.049) with significantly higher levels of hypertrophied microglia in NN versus PP (*P* = 0.007). Values represent mean ± SEM (background corrected, n = 4 to 6 per treatment). *P < 0.05, **P < 0.01; Fisher's least significant difference post-hoc analysis. Scale bar = 200 Âµm. Click here for file

Additional file 5 **Figure 5.** Reactive astrogliosis following a double immune challenge in aged non-transgenic mice. **(A-D)** Low-magnification images of immunoperoxidase staining using rabbit anti-glial fibrillary acidic protein (GFAP) antibody (AB5804) of coronal brain sections obtained from 18-month-old control mice (NN; NaCI at gestational day (GD) and 15 months), and prenatal (PN; polyriboinosinic-polyribocytidilic acid (PolyI:C) at GD17 and NaCI at 15 months), adult (NP; NaCI at GD17 and NaCI at 15 months), and double (PP, PolyI:C at GD17 and 15 months) immune-challenged mice. **(D**) A pronounced increase in the density of GFAP-positive astrocytes was evident in the hippocampal formation of double immune-challenged mice compared with control subjects. Note that also an immune challenge **(B)** prenatally and **(C)** in adulthood alone showed a trend towards elevated/reactive astrogliosis. **(E)** ANOVA yielded a main effect of treatment for the quantification of percentage area covered by anti-GFAP immunoreactivity (IR) in the hippocampus (*F*_3,16_ = 3.4, *P* = 0.040) with significant differences between NN versus PP (*P* = 0.011) and NP versus PP (*P* = 0.021). **(F)** Volumetric analysis of the hippocampus revealed no significant differences between the treatment groups at this age. Values are given as mean ± SEM, n = 4 to 6 per treatment; *P < 0.05; statistical significance based on Fisher’s least significant difference post-hoc analysis. Scale bar = 500 Âµm. Click here for file

Additional file 6 **Figure 6.** Grayscale version of the low-magnification images shown in Figure 5 (anti-Aβ_1–16_) and Figure 8 (anti-pTau^T205^). **(A,D)** Digital images obtained from NP (NaCI at GD17 and polyriboinosinic-polyribocytidilic acid (PolyI:C) at 12 months) mice. **(B,C,E)** Low-magnification and high-magnification images of brain sections taken from PP mice (PolyI:C at GD17 and 12 months). Image in panel **(C** corresponds to the boxed area in **(B)**. Scale bars **(B,E)** = 500 μm, **(C)** = 200 Âµm. Click here for file

Additional file 7 **Figure 7.** Aβ immunoreactivity (IR) in the dorsal and ventral hippocampus of 15-month-old mice. Representative images of coronal brain sections processed for immunoperoxidase staining using anti-Aβ_1–40/42_ (AB5076) antibodies. **(A-D)** Non-transgenic mice and **(E-H)** 3xTg-AD mice, harboring two transgenes (encoding APP_swe_ and TauP301L, respectively) in a homozygous PS1M146V knock-in background,received a single intravenous injection of (left lane) NaCI or (right lane) PolyI:C (5 mg/kg bodyweight) at 4 months of age. Note that the non-transgenic mice showed an increase in anti-APP/Aβ_1–40/42_ IR along the dorsal-ventral axis of the hippocampus following a single viral-like infection with **(B,D**) PolyI:C compared with **(A,C)** NaCI. **(E,G)** Control treatment of 3xTg-AD mice with NaCI during pre-plaque stage resulted in the typical appearance of Aβ plaques in the ventral subiculum, whereas **(F,H)** a single exposure to PolyI:C strongly aggravated the Aβ plaque density, covering the entire hippocampus along its septotemporal axis. Scale bars: 500 Âµm. Click here for file

Additional file 8 **Figure 8.** Summary of the semi-quantitative densitometric analysis of the anti-Aβ and anti-Tau immunoreactivity (IR). Polyriboinosinic-polyribocytidilic acid (PolyI:C; 5 mg/kg body weight) or NaCI in a injection volume of 5 ml/kg was administered intravenously to mice at 4 months of age. Brain tissue was collected at 15 months, and processed for immunoperoxidase staining. Optical-density measurements were performed in the outlined CA1 subfield (including stratum oriens, pyramidale, radiatum), lacunosum moleculare (slm), the molecular and granule cell layer of the dentate gyrus (DG), and the hilus, both in the dorsal and ventral hippocampus. Mean pixel brightness was corrected for non-specific background staining using measurements in the corpus callosum as reference, indicated as relative optical density (OD). **(A)** Summary of the changes in APP/Aβ_1–40/42_ (left) and pTau (right) levels in PolyI:C-treated compared with NaCI-treated 3xTg-AD mice. Arrows show the direction of changes in the different hippocampal subfields. Brackets indicate statistical trends, with *P* values of 0.06 to 0.09. (**B)** Summary of the APP/Aβ and pTau measurements in non-transgenic mice, contrasting PolyI:C-treated versus NaCI-treated mice. (**C**) Graphic representation of the semi-quantitative analysis of (left) the APP/Aβ_1–40/42_ and (right) pTau immunoreactivity (IR) in the dorsal and ventral hippocampus of non-transgenic and transgenic mice. Values are given as mean ± SEM; n = 4 to 7 per genotype and treatment; *P < 0.05; **P < 0.01, ANOVA and Fisher's least significant difference post-hoc test. Click here for file

Additional file 9 **Figure 9.** Various forms of human amyloid-β plaques all containing significant amounts of N-terminal proteolytic fragments of amyloid precursor protein (APP)**.** (**A-I**) Immunofluorescence staining using anti-N-APP antibodies (22C11, red), anti-Aβ_1–40/42_ (AB5076, green), and DAPI (blue) counterstaining of post-mortem brain tissue from an 88-year-old patient with AD. Paraffin sections were pretreated with (**A-F**) or without (**G-I**) formic acid (FA), with the latter allowing the detection of significant N-APP-specific immunoreactivity. Note the close association, but no colocalization, between APP ectodomains and C-terminal, Aβ-containing fragments. Scale bars = 10 Âµm. Click here for file

Additional file 10 **Figure 10.** Anti-phospho-Tau IR in the dorsal and ventral hippocampus of 15-month-old mice**.** Representative images of coronal brain sections obtained from (**A-D**) non-transgenic and (**E-H**) 3xTg-AD mice challenged with a single viral-like infection with polyriboinosinic-polyribocytidilic acid (PolyI:C) or NaCI at 4 months of age, processed for immunoperoxidase staining using anti-pTau^T205^ antibodies (Ab4841) (**A-D**) Even non-transgenic mice showed an increase in pTau IR following PolyI:C compared with NaCI injection, which was particularly prominent in interneurons in CA1 and hilar mossy cells. Asterisks indicate the position of neurons (enlarged in the inset box) with distinct somatic pTau IR. (**E,G**) 3xTg-AD mice exposed to a single intravenous NaCI infusion showed the typical phosphorylation pattern in (**E**) dorsal and (**G**) ventral CA1 pyramidal neurons. (**F,H**) A single PolyI:C v injection resulted in a significant increase in anti-pTau IR in the somatodendritic compartments compared with control treatment. (**G’-H’**) Higher magnification of the boxed area in (**G**) and (**H**)**,** respectively, showing CA1 pyramidal neurons. Scale bar: (**A-G**) = 500 μm; (**G’**) = 50 μm. Click here for file
