# Supplementary figures and images for: Systemic immune challenges trigger and drive Alzheimer-like neuropathology in mice
Source: J Neuroinflammation. 2012 Jul 2;9:151. doi: 10.1186/1742-2094-9-151 (PMC3483167; doi:10.1186/1742-2094-9-151)

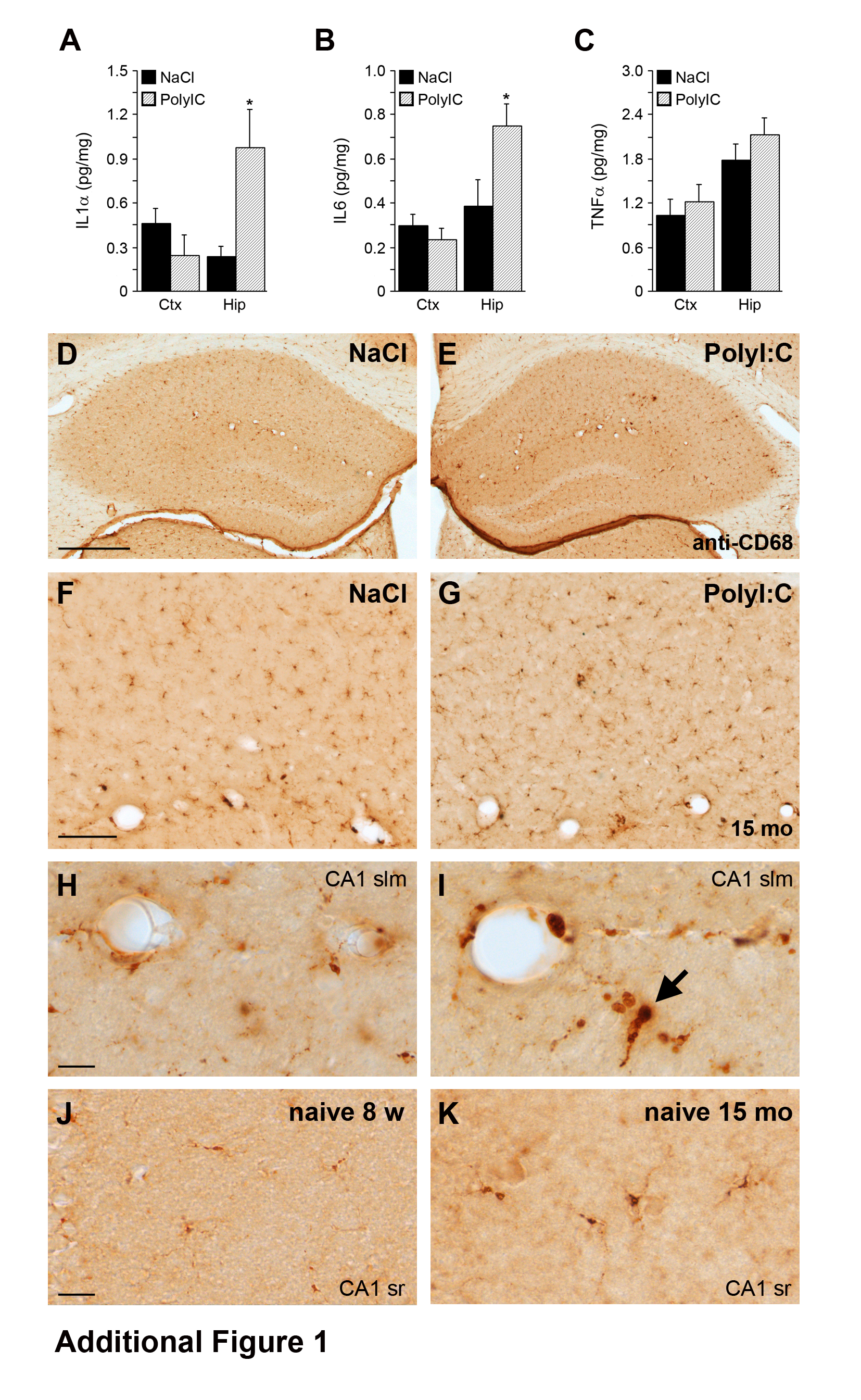

Supplement: Additional file 1 — Figure 1. Chronic increase in proinflammatory cytokines following a prenatal immune challenge in the hippocampus but not neocortex. (A-C) ELISA of neocortical (Ctx) and hippocampal (Hip) brain lysates obtained from 15-month-old polyriboinosinic-polyribocytidilic acid (PolyI:C)-treated and NaCI-treated mice (n = 4–7 per treatment group). Values represent mean ± SEM. *P < 0.05, Mann–Whitney U-test. (D-K) Immunohistochemical staining using anti-CD68 antibodies to visualize microglia in the hippocampus. Representative images are taken at different magnification from the dorsal CA1 of 15-month-old mice treated with (D,F,H) NaCI and (E,G,I) PolyI:C. Higher-magnification images show the activated stage (arrow) in microglia in the CA1 stratum lacunosum moleculare (slm) in 15-month-old mice (H) treated with PolyI:C compared with (I) control mice. (J,K) Anti-CD68 immunoreactivity (IR) in young (8 weeks) and old (15 months) naive wild-type (WT) mice. Whereas very small somata and thin processes dominated in young mice (J), microglia in old naive mice covered larger areas (K), very similar to the situation in old NaCI-exposed mice. Scale bars: (D) = 500 μm; (F) = 100 μm; (H, J) = 20 μm. [file 1742-2094-9-151-S1.tiff]

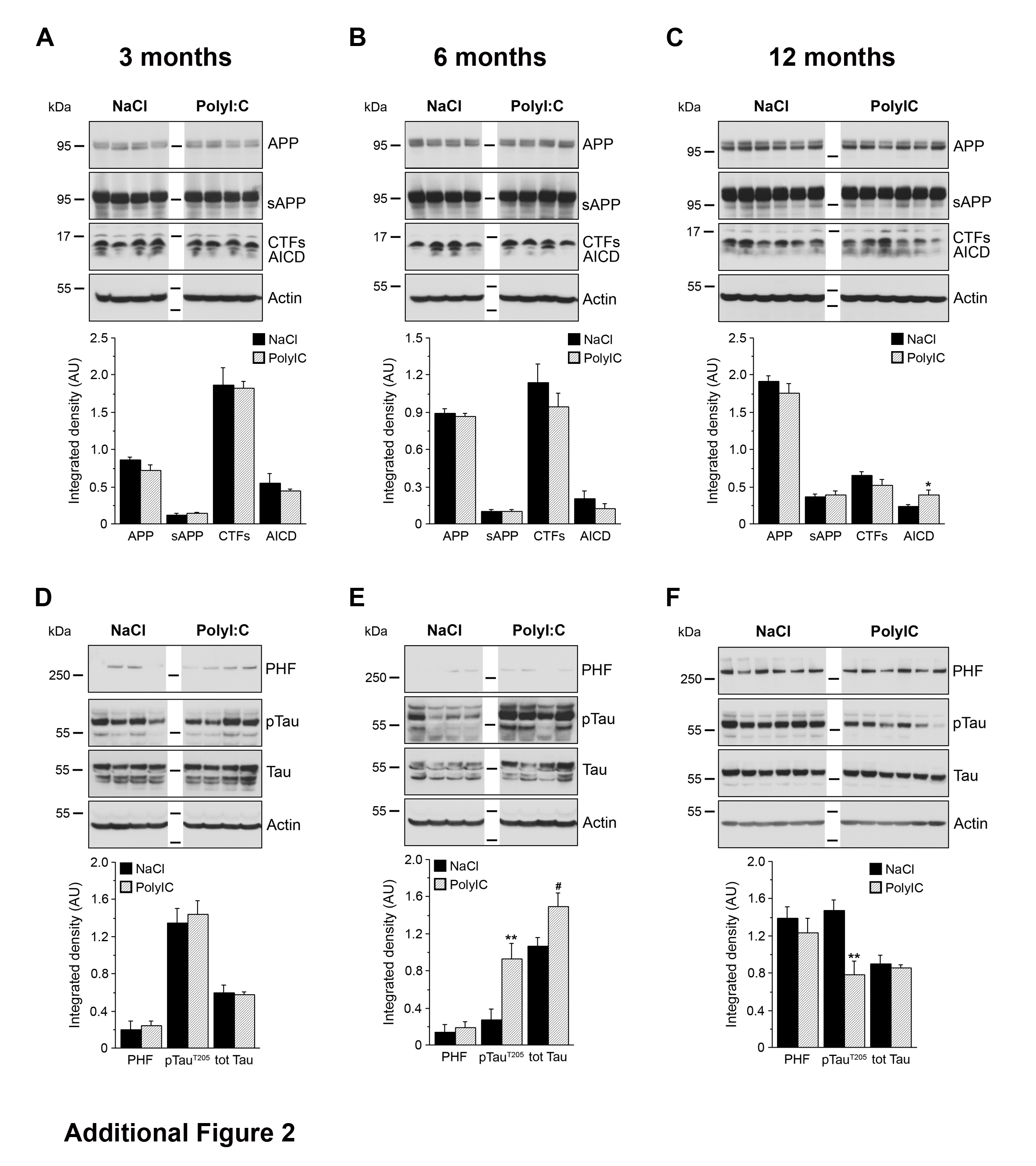

Supplement: Additional file 2 — Figure 2. Changes in amyloid precursor protein (APP) processing and Tau phosphorylation across aging after a single prenatal immune challenge in non-transgenic mice. Longitudinal study involving murine hippocampal brain lysates obtained from (A,D) 3-month-old, (B,E) 6-month-old, and (C,F) 2-month-old non-transgenic mice exposed in utero at gestation day (GD)17 to either polyriboinosinic-polyribocytidilic acid (PolyI:C) or NaCI. The latter cohort also received a single NaCI injection at 9 months, and constituted the control group for the double immune-challenged mice. Western blots were performed using the following antibodies: (A-C) mouse anti-APP A4 (clone 22 C11, recognizing full-length APP, and soluble α- or β-secretase-cleaved APP ectodomains (sAPP); rabbit anti-C-terminal APP (A8717, recognizing α- or β-secretase-generated C-terminal fragments (CTFs) and γ-secretase-cleaved APP intracellular domains (AICD)); and mouse anti-β-Actin (clone 4). (D-F) Western blots using mouse anti-paired helical filaments (PHFs, clone AT100), rabbit anti-phosphorylated Tau205, and mouse anti-total Tau (Tau-5) antibodies. Quantitative analysis involved the measurement of the integrated pixel brightness of the immunoreactive bands, corrected for non-specific background and equal loading using β-actin as control. Please note that the pTau blot in E has been overexposed for visual display. Western blot lanes show the different subjects. Values represent mean relative, β-actin-corrected optical density expressed in arbitrary units (AU) (mean ± SEM, n = 4 to 6 per treatment and age). *P < 0.05, **P < 0.01, # P = 0.053, Mann–Whitney U-test. [file 1742-2094-9-151-S2.tiff]

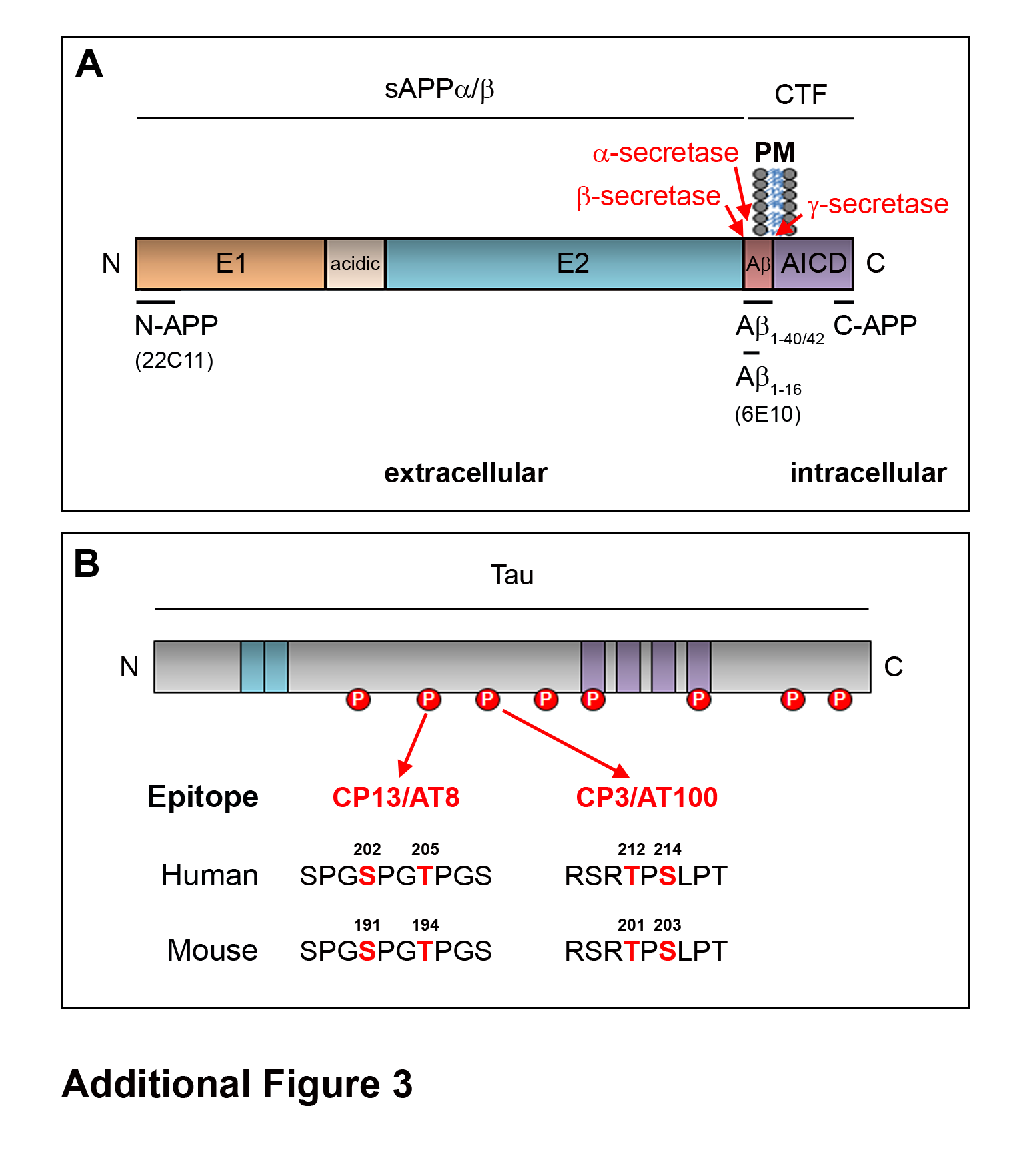

Supplement: Additional file 3 — Figure 3. (A) Schematic representation of the domain structure of the amyloid precursor protein (short form, APP695), indicatingα-, β- and γ-secretase cleavage sites and antibody binding sites. Note that antibody Aβ1–16 (6E10) recognizes both Aβ/C-terminal fragments (CTFs) as well as full-length APP on western blots and tissue sections (see Figure 5). By contrast, antibody Aβ1–40/42 has very low affinity for full-length APP but strongly binds to Aβ/CTF fragments. (B) Schematic representation of the human Tau protein (441 amino acid, longest isoform). Alternative splicing of mRNA from a single gene located on the long arm of chromosome 17 yields six different isoforms that are expressed in the adult human brain [62]. They differ by the presence of one to two amino-terminal inserts (blue) and three to four tandem repeats (purple) in the carboxyterminal region. The sequence common to all known isoforms is shown in gray. The phosphorylation-dependent anti-Tau antibodies used are listed (including their alternative names) along with the respective phosphorylation-site and flanking sequences. As can be seen from the alignment of the tau sequences in five species, there is a high level of homology between mouse and human tau. Note that pTauT205 antibody also crossreacts with Ser199 in mouse Tau, an epitope that can also be detected with the AT100 (PHF) antibody [63] Abbreviations:CTF, C-terminal fragment; AICD, APP intracellular domain; PM, plasma membrane; sAPPα/β,soluble α- or β-secretase-cleaved. [file 1742-2094-9-151-S3.tiff]

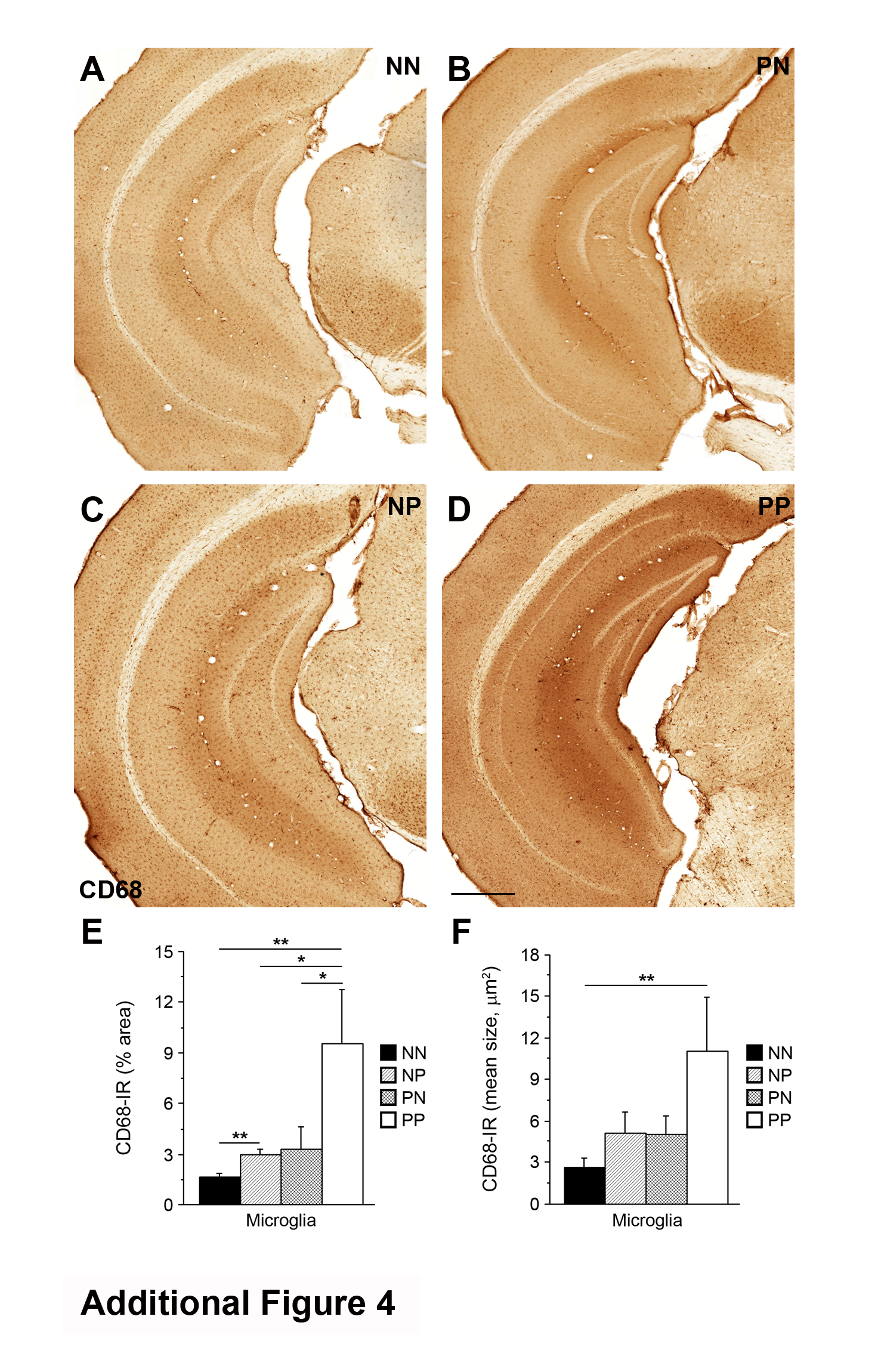

Supplement: Additional file 4 — Figure 4. Microglia responses in double immune-challenged non-transgenic mice. (A-D) Low-magnification images of immunoperoxidase staining using anti-CD68 antibody (MCA 341R) of coronal brain sections obtained from 18-month-old control mice (NN; NaCI at gestational day (GD) and 15 months), and prenatal (PN; polyriboinosinic-polyribocytidilic acid (PolyI:C) at GD17 and NaCI at 15 months), adult (NP; NaCI at GD17 and PolyI:C at 15 months), and double (PP, PolyI:C at GD17 and 15 months) immune-challenged mice. (D) A pronounced increase in the density of activated CD68-positive microglia with hypertrophied and ameboid morphology was found throughout the hippocampal formation of double immune-challenged mice compared with control subjects. Note that also immune challenge (B) prenatally and (C) in adulthood alone had a stimulating effect on microglia OD. (E-F) Quantitative analysis of the immunoreactive signals labeled with antibodies against rat CD68. (E) ANOVA yielded a main effect of treatment for the quantification of percentage area covered by anti-CD68 immunoreactivity (IR) in the hippocampus (F3,16 = 4.4, P = 0.019) with significant differences between NN versus PP (P = 0.004), NP versus PP (P = 0.015), and PN versus PP (P = 0.026). (F) A significant main effect also emerged for the mean area (F3,16 = 3.2, P = 0.049) with significantly higher levels of hypertrophied microglia in NN versus PP (P = 0.007). Values represent mean ± SEM (background corrected, n = 4 to 6 per treatment). *P < 0.05, **P < 0.01; Fisher's least significant difference post-hoc analysis. Scale bar = 200 Âµm. [file 1742-2094-9-151-S4.tiff]

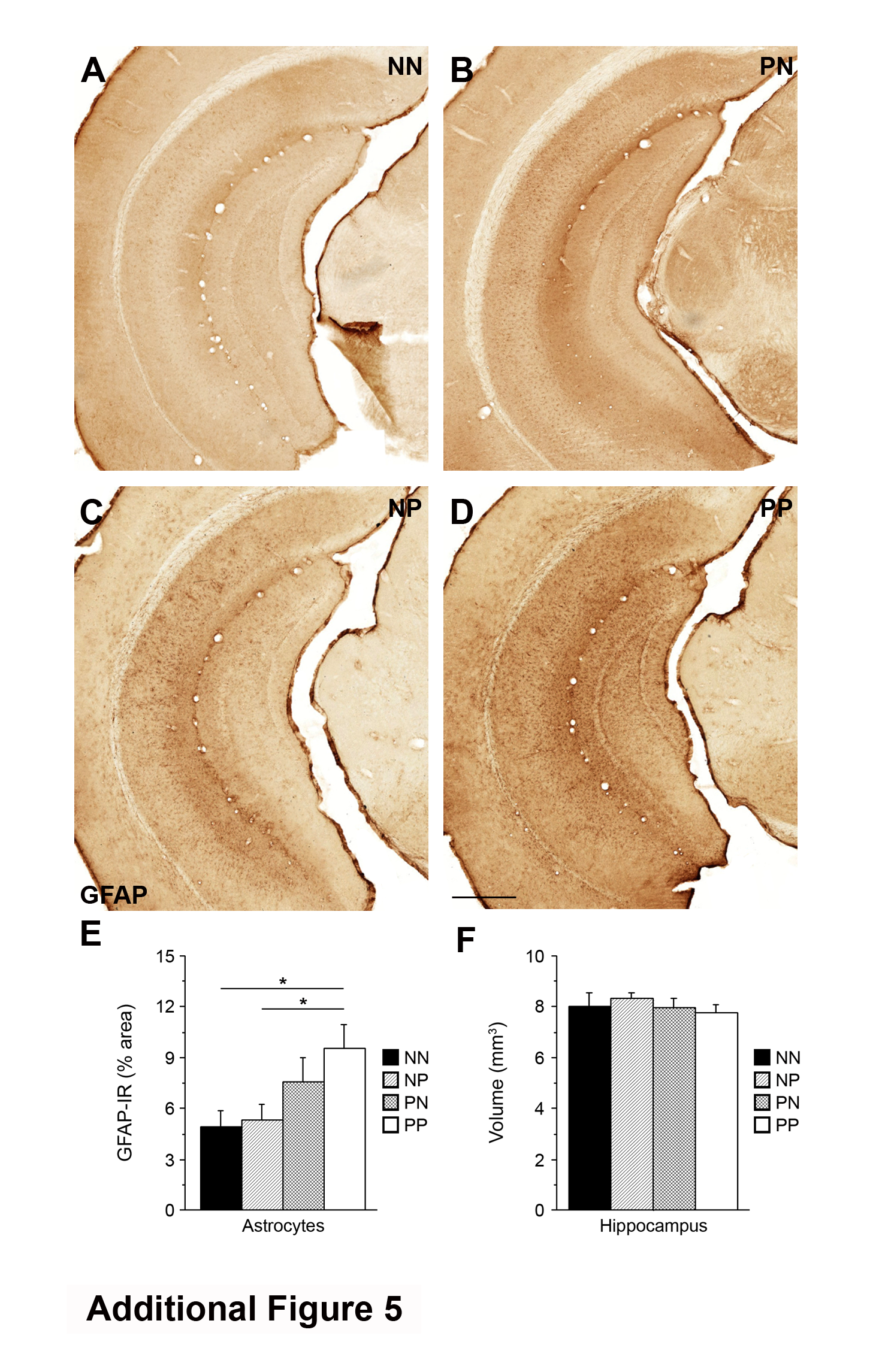

Supplement: Additional file 5 — Figure 5. Reactive astrogliosis following a double immune challenge in aged non-transgenic mice. (A-D) Low-magnification images of immunoperoxidase staining using rabbit anti-glial fibrillary acidic protein (GFAP) antibody (AB5804) of coronal brain sections obtained from 18-month-old control mice (NN; NaCI at gestational day (GD) and 15 months), and prenatal (PN; polyriboinosinic-polyribocytidilic acid (PolyI:C) at GD17 and NaCI at 15 months), adult (NP; NaCI at GD17 and NaCI at 15 months), and double (PP, PolyI:C at GD17 and 15 months) immune-challenged mice. (D) A pronounced increase in the density of GFAP-positive astrocytes was evident in the hippocampal formation of double immune-challenged mice compared with control subjects. Note that also an immune challenge (B) prenatally and (C) in adulthood alone showed a trend towards elevated/reactive astrogliosis. (E) ANOVA yielded a main effect of treatment for the quantification of percentage area covered by anti-GFAP immunoreactivity (IR) in the hippocampus (F3,16 = 3.4, P = 0.040) with significant differences between NN versus PP (P = 0.011) and NP versus PP (P = 0.021). (F) Volumetric analysis of the hippocampus revealed no significant differences between the treatment groups at this age. Values are given as mean ± SEM, n = 4 to 6 per treatment; *P < 0.05; statistical significance based on Fisher’s least significant difference post-hoc analysis. Scale bar = 500 Âµm. [file 1742-2094-9-151-S5.tiff]

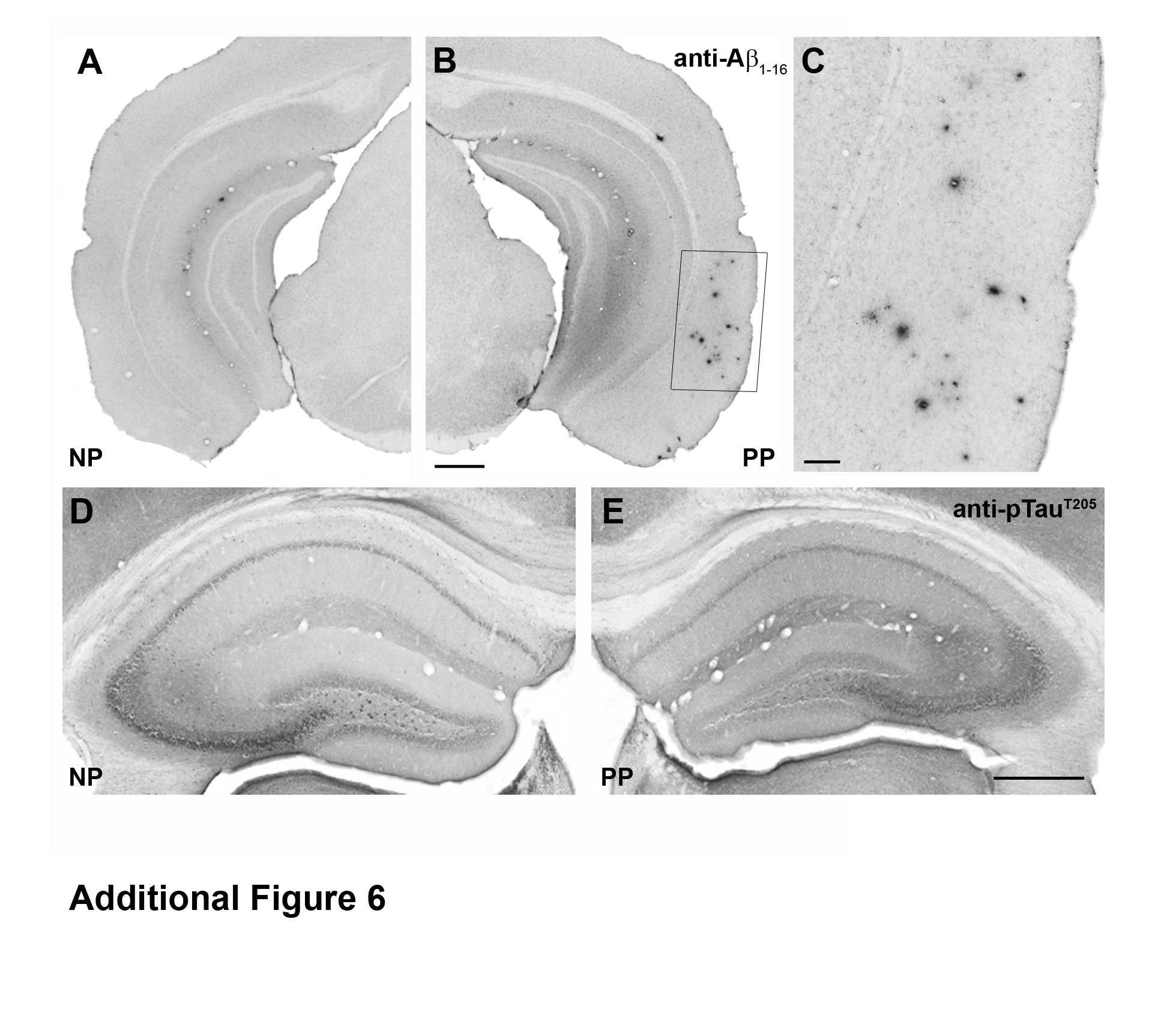

Supplement: Additional file 6 — Figure 6. Grayscale version of the low-magnification images shown in Figure 5 (anti-Aβ1–16) and Figure 8 (anti-pTauT205). (A,D) Digital images obtained from NP (NaCI at GD17 and polyriboinosinic-polyribocytidilic acid (PolyI:C) at 12 months) mice. (B,C,E) Low-magnification and high-magnification images of brain sections taken from PP mice (PolyI:C at GD17 and 12 months). Image in panel (C corresponds to the boxed area in (B). Scale bars (B,E) = 500 μm, (C) = 200 Âµm. [file 1742-2094-9-151-S6.tiff]

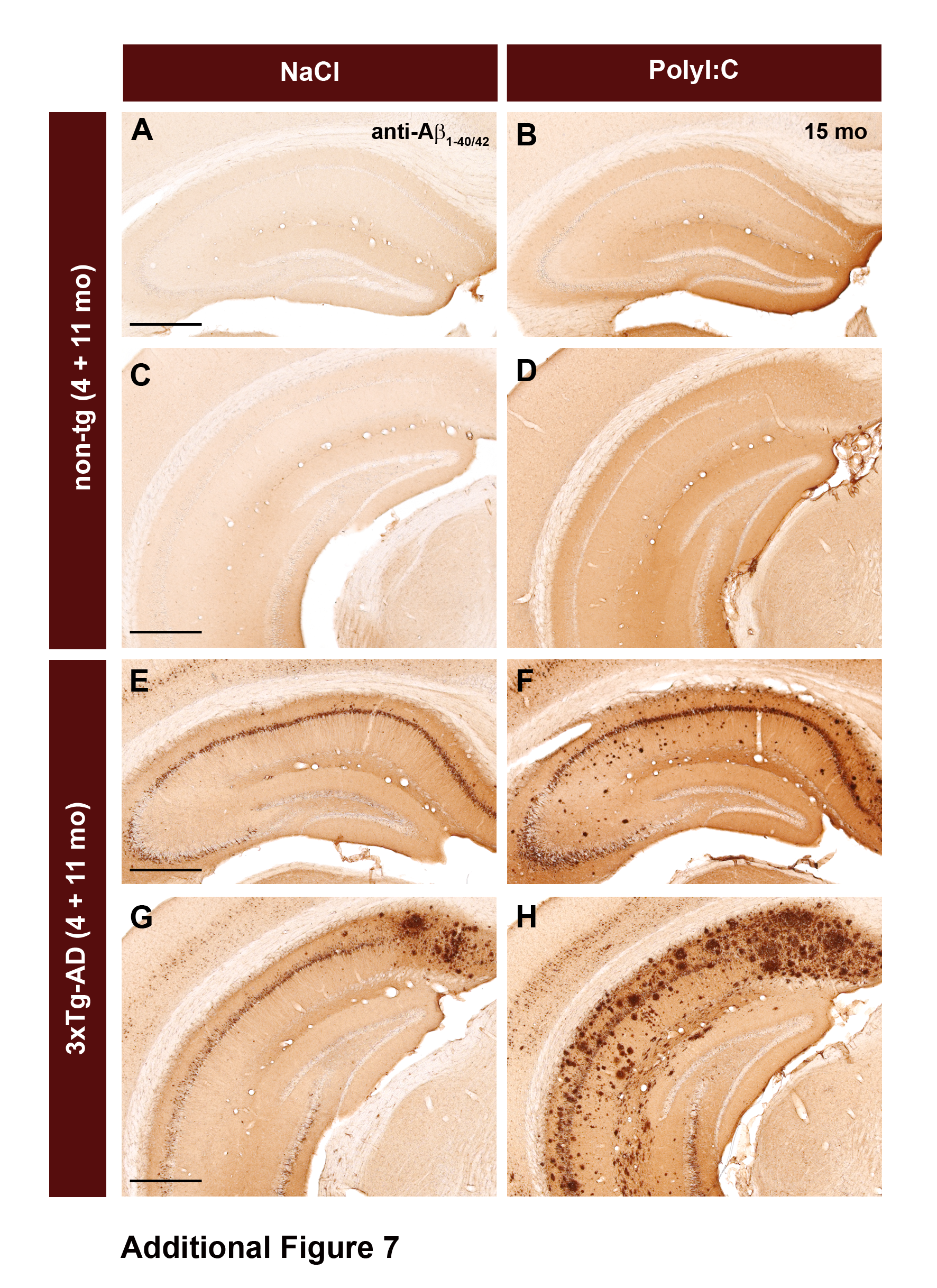

Supplement: Additional file 7 — Figure 7. Aβ immunoreactivity (IR) in the dorsal and ventral hippocampus of 15-month-old mice. Representative images of coronal brain sections processed for immunoperoxidase staining using anti-Aβ1–40/42 (AB5076) antibodies. (A-D) Non-transgenic mice and (E-H) 3xTg-AD mice, harboring two transgenes (encoding APPswe and TauP301L, respectively) in a homozygous PS1M146V knock-in background,received a single intravenous injection of (left lane) NaCI or (right lane) PolyI:C (5 mg/kg bodyweight) at 4 months of age. Note that the non-transgenic mice showed an increase in anti-APP/Aβ1–40/42 IR along the dorsal-ventral axis of the hippocampus following a single viral-like infection with (B,D) PolyI:C compared with (A,C) NaCI. (E,G) Control treatment of 3xTg-AD mice with NaCI during pre-plaque stage resulted in the typical appearance of Aβ plaques in the ventral subiculum, whereas (F,H) a single exposure to PolyI:C strongly aggravated the Aβ plaque density, covering the entire hippocampus along its septotemporal axis. Scale bars: 500 Âµm. [file 1742-2094-9-151-S7.tiff]

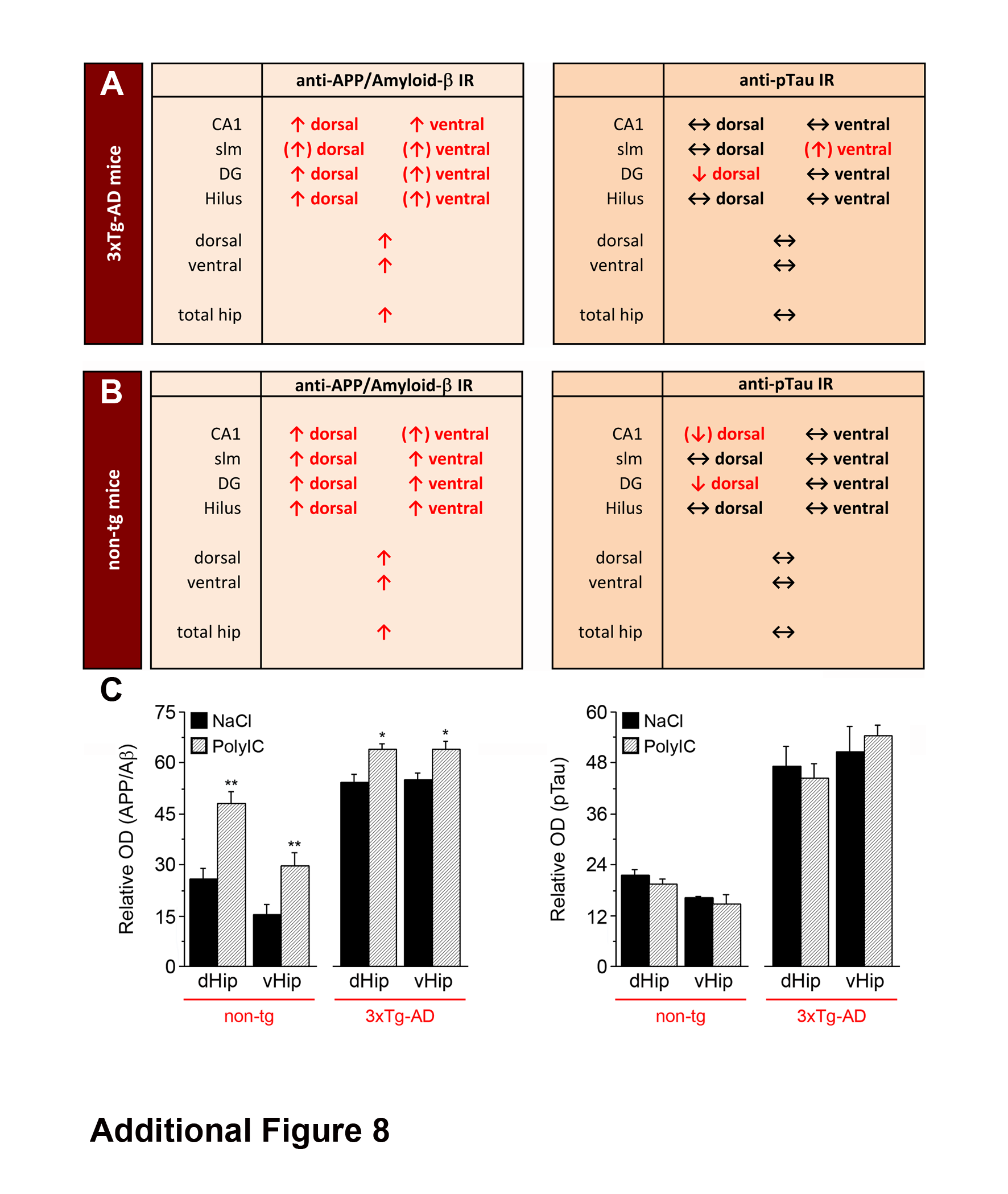

Supplement: Additional file 8 — Figure 8. Summary of the semi-quantitative densitometric analysis of the anti-Aβ and anti-Tau immunoreactivity (IR). Polyriboinosinic-polyribocytidilic acid (PolyI:C; 5 mg/kg body weight) or NaCI in a injection volume of 5 ml/kg was administered intravenously to mice at 4 months of age. Brain tissue was collected at 15 months, and processed for immunoperoxidase staining. Optical-density measurements were performed in the outlined CA1 subfield (including stratum oriens, pyramidale, radiatum), lacunosum moleculare (slm), the molecular and granule cell layer of the dentate gyrus (DG), and the hilus, both in the dorsal and ventral hippocampus. Mean pixel brightness was corrected for non-specific background staining using measurements in the corpus callosum as reference, indicated as relative optical density (OD). (A) Summary of the changes in APP/Aβ1–40/42 (left) and pTau (right) levels in PolyI:C-treated compared with NaCI-treated 3xTg-AD mice. Arrows show the direction of changes in the different hippocampal subfields. Brackets indicate statistical trends, with P values of 0.06 to 0.09. (B) Summary of the APP/Aβ and pTau measurements in non-transgenic mice, contrasting PolyI:C-treated versus NaCI-treated mice. (C) Graphic representation of the semi-quantitative analysis of (left) the APP/Aβ1–40/42 and (right) pTau immunoreactivity (IR) in the dorsal and ventral hippocampus of non-transgenic and transgenic mice. Values are given as mean ± SEM; n = 4 to 7 per genotype and treatment; *P < 0.05; **P < 0.01, ANOVA and Fisher's least significant difference post-hoc test. [file 1742-2094-9-151-S8.tiff]

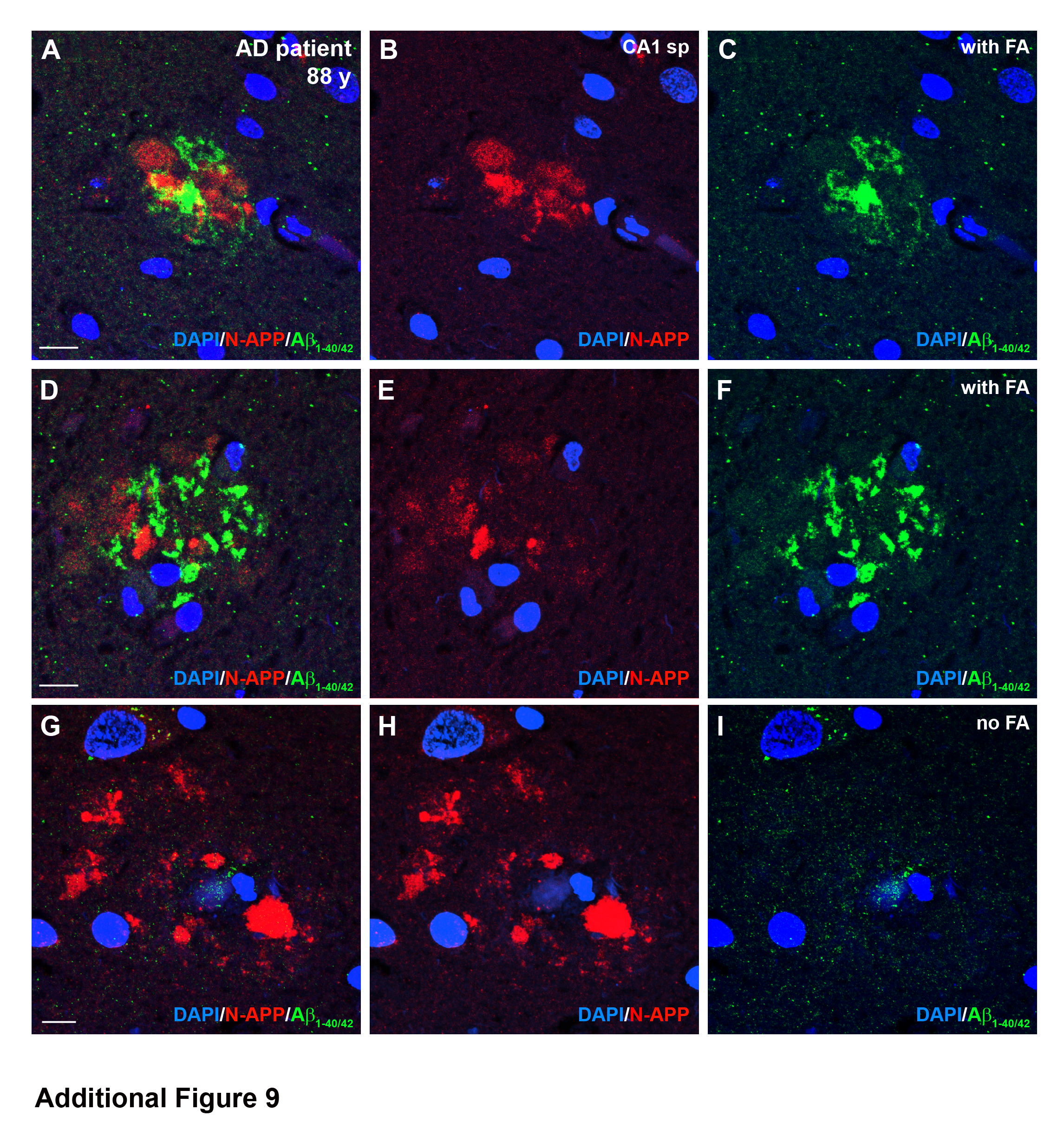

Supplement: Additional file 9 — Figure 9. Various forms of human amyloid-β plaques all containing significant amounts of N-terminal proteolytic fragments of amyloid precursor protein (APP). (A-I) Immunofluorescence staining using anti-N-APP antibodies (22C11, red), anti-Aβ1–40/42 (AB5076, green), and DAPI (blue) counterstaining of post-mortem brain tissue from an 88-year-old patient with AD. Paraffin sections were pretreated with (A-F) or without (G-I) formic acid (FA), with the latter allowing the detection of significant N-APP-specific immunoreactivity. Note the close association, but no colocalization, between APP ectodomains and C-terminal, Aβ-containing fragments. Scale bars = 10 Âµm. [file 1742-2094-9-151-S9.tiff]

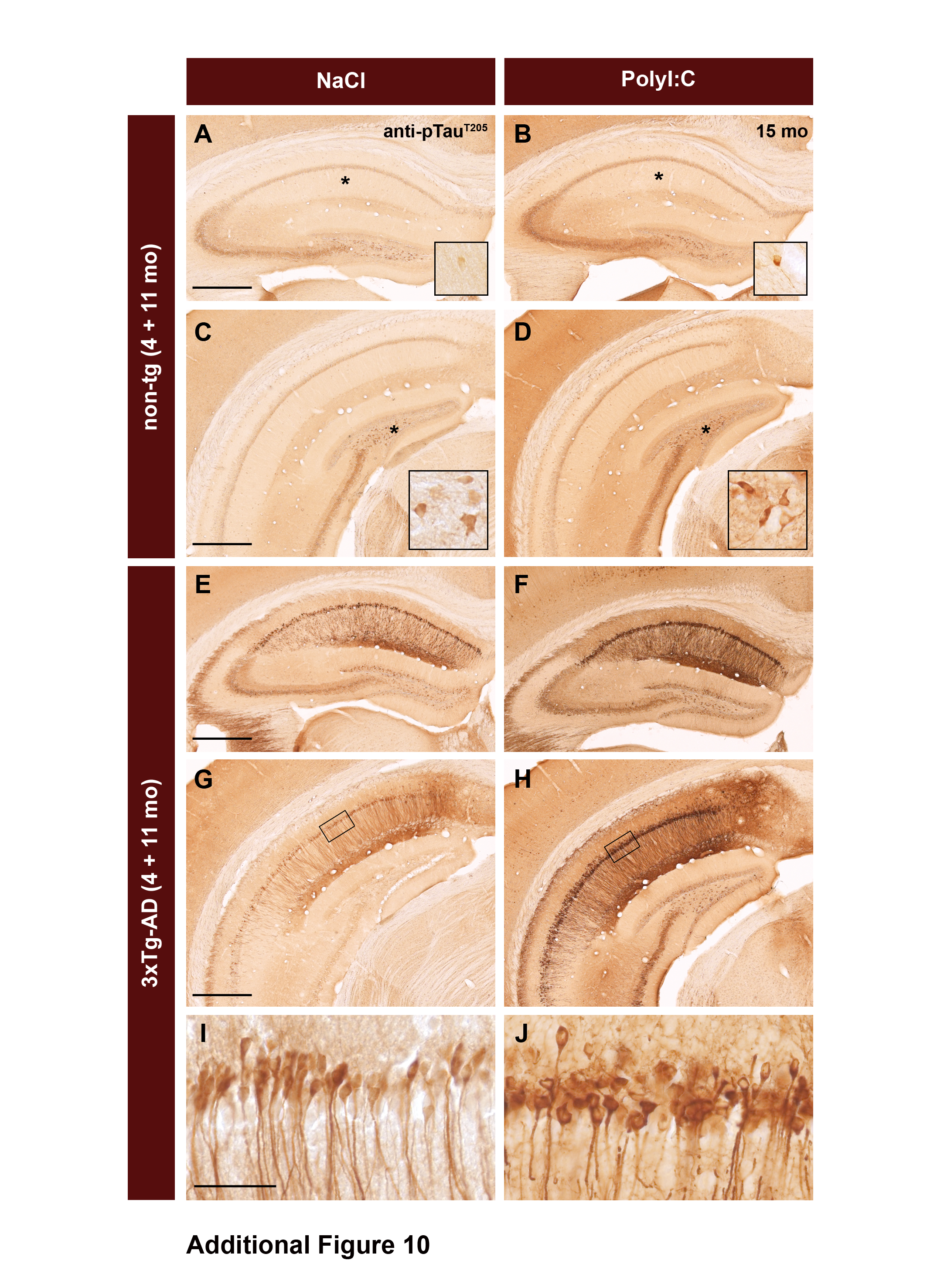

Supplement: Additional file 10 — Figure 10. Anti-phospho-Tau IR in the dorsal and ventral hippocampus of 15-month-old mice. Representative images of coronal brain sections obtained from (A-D) non-transgenic and (E-H) 3xTg-AD mice challenged with a single viral-like infection with polyriboinosinic-polyribocytidilic acid (PolyI:C) or NaCI at 4 months of age, processed for immunoperoxidase staining using anti-pTauT205 antibodies (Ab4841) (A-D) Even non-transgenic mice showed an increase in pTau IR following PolyI:C compared with NaCI injection, which was particularly prominent in interneurons in CA1 and hilar mossy cells. Asterisks indicate the position of neurons (enlarged in the inset box) with distinct somatic pTau IR. (E,G) 3xTg-AD mice exposed to a single intravenous NaCI infusion showed the typical phosphorylation pattern in (E) dorsal and (G) ventral CA1 pyramidal neurons. (F,H) A single PolyI:C v injection resulted in a significant increase in anti-pTau IR in the somatodendritic compartments compared with control treatment. (G’-H’) Higher magnification of the boxed area in (G) and (H), respectively, showing CA1 pyramidal neurons. Scale bar: (A-G) = 500 μm; (G’) = 50 μm. [file 1742-2094-9-151-S10.tiff]
